# A fully automated and explainable algorithm for predicting malignant transformation in oral epithelial dysplasia

**DOI:** 10.1038/s41698-024-00624-8

**Published:** 2024-06-28

**Authors:** Adam J. Shephard, Raja Muhammad Saad Bashir, Hanya Mahmood, Mostafa Jahanifar, Fayyaz Minhas, Shan E. Ahmed Raza, Kris D. McCombe, Stephanie G. Craig, Jacqueline James, Jill Brooks, Paul Nankivell, Hisham Mehanna, Syed Ali Khurram, Nasir M. Rajpoot

**Affiliations:** 1 Tissue Image Analytics Centre, Department of Computer Science, University of Warwick, Coventry, UK; 2 School of Clinical Dentistry, University of Sheffield, Sheffield, UK; 3 Precision Medicine Centre, Patrick G. Johnston Centre for Cancer Research, Queen’s University Belfast, Belfast, UK; 4 Institute of Head and Neck Studies and Education, Institute of Cancer and Genomic Sciences, University of Birmingham, Birmingham, UK

**Keywords:** Oral cancer detection, Mathematics and computing

## Abstract

Oral epithelial dysplasia (OED) is a premalignant histopathological diagnosis given to lesions of the oral cavity. Its grading suffers from significant inter-/intra-observer variability, and does not reliably predict malignancy progression, potentially leading to suboptimal treatment decisions. To address this, we developed an artificial intelligence (AI) algorithm, that assigns an Oral Malignant Transformation (OMT) risk score based on the Haematoxylin and Eosin (H&E) stained whole slide images (WSIs). Our AI pipeline leverages an in-house segmentation model to detect and segment both nuclei and epithelium. Subsequently, a shallow neural network utilises interpretable morphological and spatial features, emulating histological markers, to predict progression. We conducted internal cross-validation on our development cohort (Sheffield; *n* = 193 cases) and independent validation on two external cohorts (Birmingham and Belfast; *n* = 89 cases). On external validation, the proposed *OMTscore* achieved an AUROC = 0.75 (Recall = 0.92) in predicting OED progression, outperforming other grading systems (Binary: AUROC = 0.72, Recall = 0.85). Survival analyses showed the prognostic value of our *OMTscore* (C-index = 0.60, *p* = 0.02), compared to WHO (C-index = 0.64, *p* = 0.003) and binary grades (C-index = 0.65, *p* < 0.001). Nuclear analyses elucidated the presence of peri-epithelial and intra-epithelial lymphocytes in highly predictive patches of transforming cases (*p* < 0.001). This is the first study to propose a completely automated, explainable, and externally validated algorithm for predicting OED transformation. Our algorithm shows comparable-to-human-level performance, offering a promising solution to the challenges of grading OED in routine clinical practice.

## Introduction

Head and neck cancer is among the top ten most prevalent cancers globally^1^, constituting a significant public health challenge. In Europe alone, approximately 150,000 new cases are reported annually^2^. These cancers are often detected at an advanced stage (approximately 60%), resulting in poor prognosis and a five-year survival rate of only 40%^2^. With early diagnosis followed by timely treatment, survival increases to 80-90%^2^. Therefore, early detection plays a crucial role in improving patient outcomes.

Oral squamous cell carcinoma (OSCC) is the most common type of head and neck cancer^1^, that may arise from an oral potentially malignant disorder (OPMD) such as leukoplakia or erythroplakia^3^. These disorders are often associated with lifestyle habits such as tobacco smoking, betel quid chewing, and excessive alcohol consumption, although genetic factors may also play a role^4–6^. Following a biopsy and microscopic examination, these lesions may be given a histopathological diagnosis of oral epithelial dysplasia (OED), which carries a higher risk of progressing to OSCC^4,7^. Histological atypia in OED typically manifests in the basal layer and progresses upwards through the epithelial layers. Cytological changes often include changes to the shape, size, and colour of nuclei/cells, the presence of atypical mitotic figures, and increased cellularity^3^. Architectural changes typically include irregular epithelial stratification, loss of basal cell polarity, drop-shaped rete pegs, and loss of epithelial cohesion^3^.

There are different grading systems to classify OED and inform treatment decisions. The 2017 World Health Organisation (WHO) grading is a three-tier system for grading cases as mild, moderate, and severe, taking into account over 15 different features. This system splits the epithelium into thirds, suggesting that architectural/cytological changes confined to the lower third may be classed as mild, in the middle moderate, and those progressing towards the upper third as severe^8^. However, this system oversimplifies a complex disease process, lacks standardisation, and introduces ambiguity and subjectivity, which could result in an inaccurate diagnosis with potentially detrimental implications for outcomes. A meta-analysis conducted by Iocca et al.^9^, confirmed the greater risk of malignant transformation in moderate/severe dysplasia cases when compared to mild cases. An alternate binary grading system, categorising lesions as low- or high-risk, based on the number of cytological and architectural features, aimed to improve grade reproducibility^8,10^. However, studies have shown significant variability in grading using both systems^3^, highlighting the need for a more objective and reproducible method that can better predict malignant transformation in OED.

The availability of graphical processing units (GPU) and the rise of convolutional neural networks (CNNs) and deep learning have revolutionised computer vision, including medical imaging^11^. Computational pathology is an active area of research that leverages machine learning and deep learning algorithms for the analysis of histological patterns in multi-gigapixel whole-slide images (WSIs) to tackle pathology-related tasks^12,13^. Deep learning models have become commonplace in laboratories worldwide, being used for tasks such as segmentation, detection, and classification^14–18^. Numerous deep learning algorithms have been applied to tasks such as tissue and nuclei segmentation in WSIs^19–24^, as well as making slide-level predictions for histopathological diagnoses^25–27^. Multiple studies have proposed generating slide-level predictions by aggregating patch-level predictions or features using pooling or attention-based mechanisms^28–33^. Efforts are underway to consolidate the diverse deep learning methods employed in computational pathology, exemplified by initiatives like the *TIAToolbox*^34^.

Several studies have explored the use of artificial intelligence (AI) in grading and prognostication of OED lesions. Bashir et al.^23^ used the mean widths of epithelial layers as a proxy for epithelial stratification, within Random Forests to predict OED grade. Shephard et al.^26^ achieved varying success in predicting OED recurrence/transformation using nuclear shape/size features in H&E images. Mahmood et al.^35^ employed pathologist-derived features in Cox proportional hazards regression models to predict recurrence and transformation, identifying prognostic features such as bulbous rete pegs, hyperchromatism, and nuclear pleomorphism. Although manual feature extraction was required, the study demonstrated the link between OED features and clinical outcome. In contrast, Bashir et al.^36^ used weakly supervised multiple instance learning and identified peri-epithelial lymphocytes (PELs) as a prognostic feature for transformation at the WSI-level. However, this method required manually refined epithelial masks, and its success was not validated on external datasets. These studies demonstrate the potential of AI in improving OED diagnosis and prognosis but also emphasise the need for further development and validation of fully automated methods.

In this study, we present an end-to-end, fully automated and explainable pipeline for predicting OED transformation. We utilise an in-house multi-task model^20^ to generate nuclear and intra-epithelial layer segmentations and extract morphological/spatial features. These features are then fed into a multi-layer perceptron (MLP) to predict slide-level malignant transformation of OED. Our contributions to the scientific community include:

1. Introduction of our pipeline’s automatically generated *OMTscore*, to improve diagnostic OED grading. External validation of the *OMTscore* was conducted on independent cohorts from Birmingham and Belfast, UK.
2. Presentation of a newly trained HoVer-Net+, a state-of-the-art model capable of simultaneous segmentation and classification of nuclear instances and intra-epithelial layers. We have released the model code and weights as part of the TIAToolbox^34^, along with an example notebook (https://github.com/TissueImageAnalytics/tiatoolbox/blob/develop/examples/09-multi-task-segmentation.ipynb).
3. Demonstrated the capability of our *OMTscore* when compared to conventional histological grading in predicting malignancy transformation. Our code for model inference is publicly accessible at: https://github.com/adamshephard/OMTscoring_inference.

## Results

**Fig. 1:**
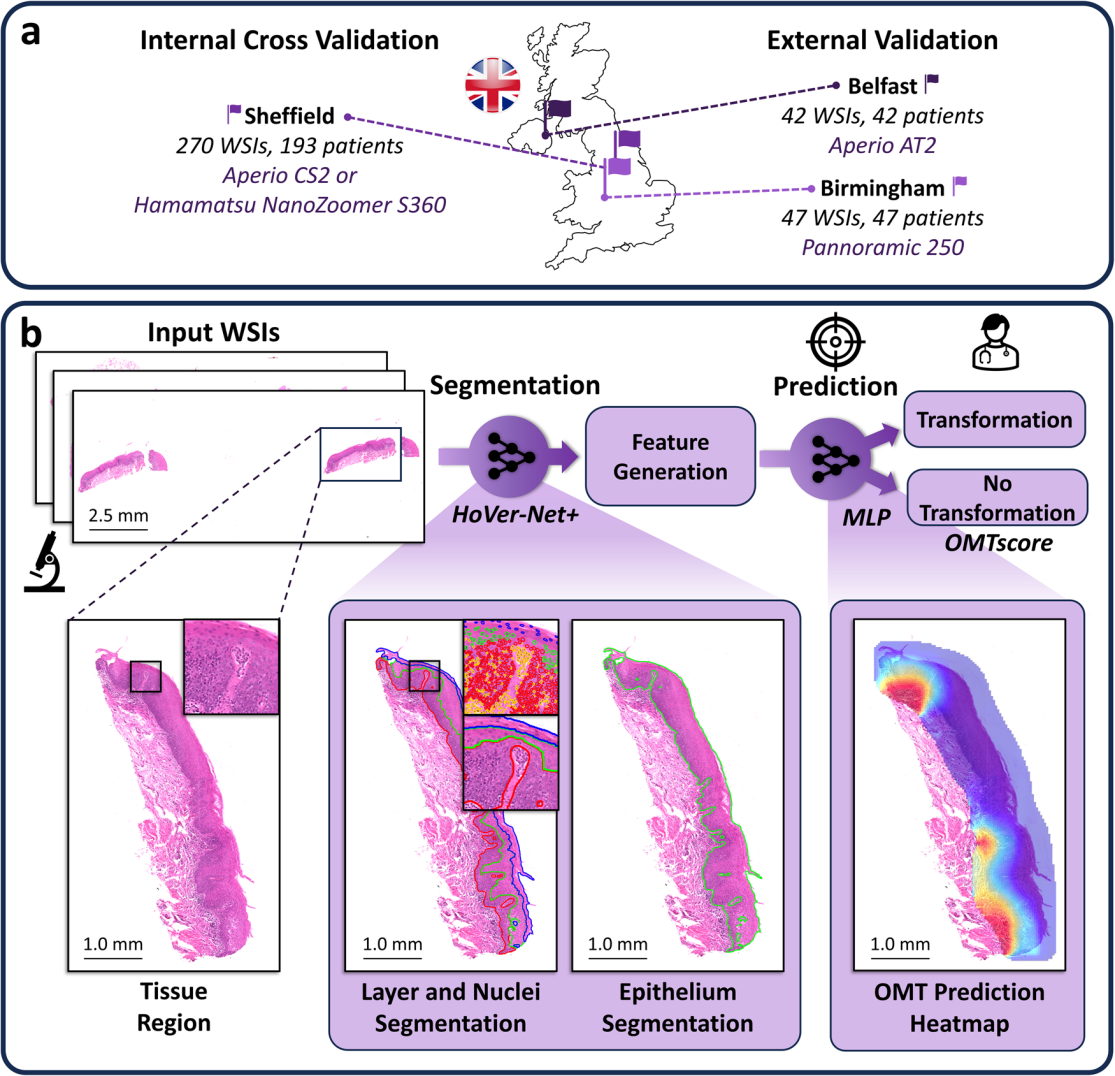
Proposed pipeline for generating the *OMTscore* for predicting malignant transformation. **a** Overview of the data used in our experiments from three different centres. This includes Sheffield data for internal training/validation, and Birmingham and Belfast data for external validation. **b** Summary of the model pipeline for generating an *OMTscore*. The model first uses a newly trained HoVer-Net+ to generate nuclear and layer segmentations. Next, patch-level morphological/spatial features are generated, and used within a trained MLP for predicting malignant transformation (i.e. the *OMTscore)*.

To predict the OED risk score (*OMTscore*), we implemented a multi-step pipeline (see Fig. 1). First, we trained an in-house deep learning model for the segmentation of both intra-epithelial layers and nuclei. We then used the trained model to produce segmentations for all slides in our cohorts. Following this, we tessellated each slide into tiles and generated tile-level morphological features (based on these nuclear segmentations) for tiles within the epithelium. Finally, these tile-level features were used within an MLP to predict whether the case transformed to malignancy (our *OMTscore*).

### Layer and nuclei segmentation

The first stage of our model pipeline involved generating both nuclear and epithelium segmentation masks for all WSIs in our internal and external cohorts. We perform this task simultaneously with HoVer-Net+^20^, a multi-task model that takes input H&E-stained images and produces nuclear instance segmentations (and classifications) and intra-epithelial layer segmentation maps. We trained and evaluated our model’s segmentation performance based on the internal Sheffield dataset alone. For an overview of the model performance for semantic segmentation and nuclear segmentation/classification, see Table 1. For a visual comparison between HoVer-Net+ results and ground-truth annotations, refer to Supplementary Fig. 2. Overall, we deemed these results satisfactory and thus used the trained HoVer-Net+ model for inference on cases from both internal and external cohorts.

**Table 1:** Performance metrics for HoVer-Net+ on internal testing

Semantic Segmentation, F1		Nuclear Segmentation		Nuclear Classification, F1_c_	
Background	0.88	Dice	0.69	Other	0.72
Other Tissue	0.84	AJI	0.62	Basal Epithelium	0.61
Basal Epithelium	0.74	DQ	0.74	Epithelium	0.66
Epithelium	0.87	SQ	0.69	Mean	0.66
Keratin	0.81	PQ	0.51		
Mean	0.83	F1_d_	0.82		

The provided Dice score is for nuclei vs background. *AJI* Aggregated Jaccard Index, *DQ* Detection Quality, *SQ* Segmentation Quality, *PQ* Panoptic Quality, *F1*_*d*_ F1-score for detection over all nuclear types, *F1*_*c*_ F1-score for classification.

### Slide-level transformation prediction

After segmentation, each WSI was tessellated into smaller 512 × 512 tiles (20× magnification, 0.50 microns per pixel, mpp), and tile-level features were generated, based on the HoVer-Net+ nuclear segmentations. For slide-level prediction, an MLP was trained using the iterative draw-and-rank method introduced by Bilal et al.^29^ with our tile-level features. We call the output of our MLP model, the *OMTscore*.

**Fig. 2:**
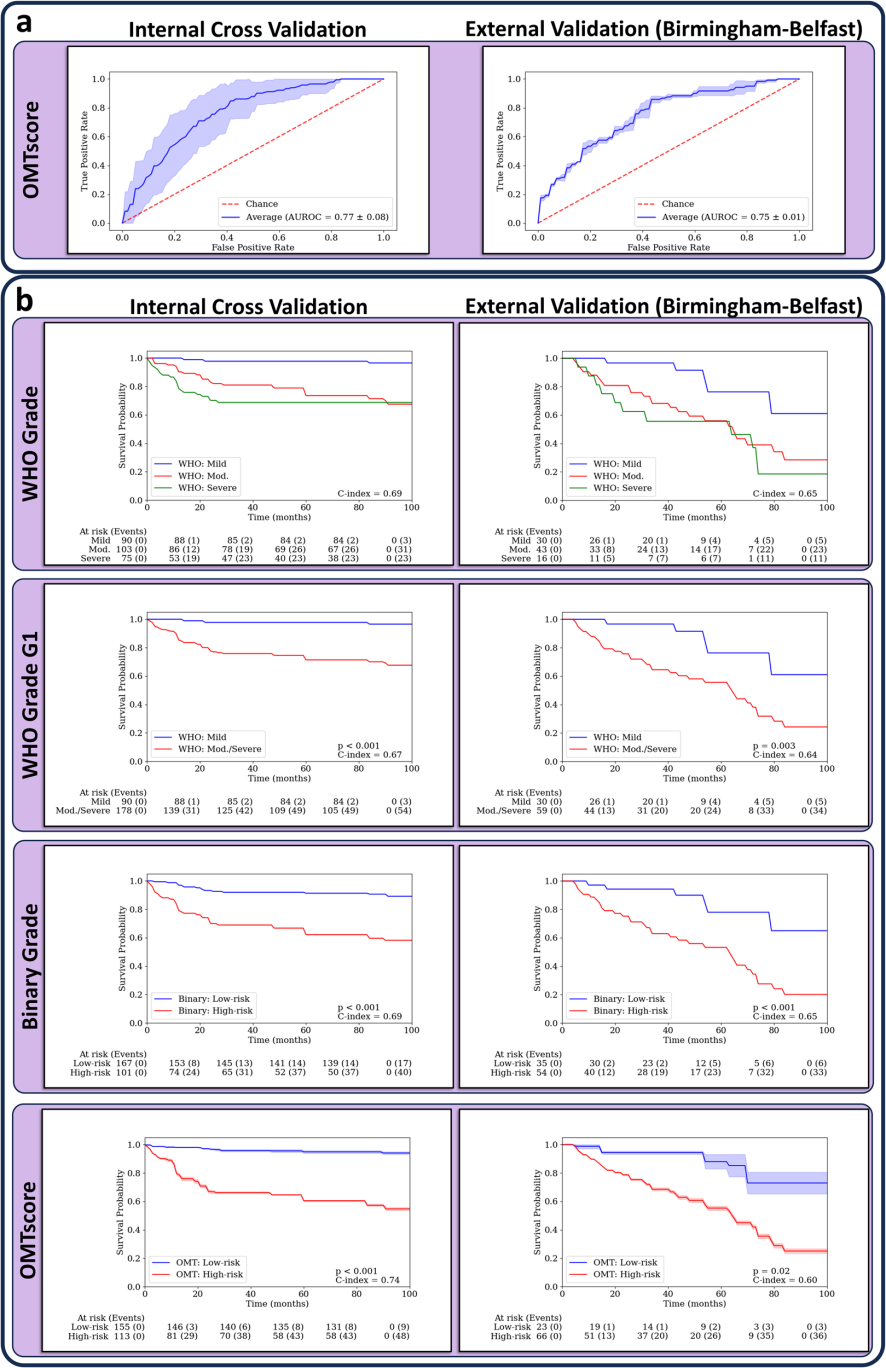
ROC plots and Kaplan-Meier survival curves for the *OMTscore* and pathologist grades. **a** ROC plots for predicting malignant transformation with internal validation on Sheffield (left), and external validation on the Birmingham-Belfast datasets by our algorithm (right). **b** Kaplan-Meier transformation-free survival curves based on the internal validation (left) and the external validation set (right) for the predictions from the WHO 2017 grade (top), WHO grade G1 (mild vs. moderate/severe), the binary grade and the *OMTscore* (bottom). Confidence intervals supplied for the *OMTscore* output AUROC/Kaplan-Meier curves are generated by the standard deviation of the model output over repeated runs of the experiment.

**Fig. 3:**
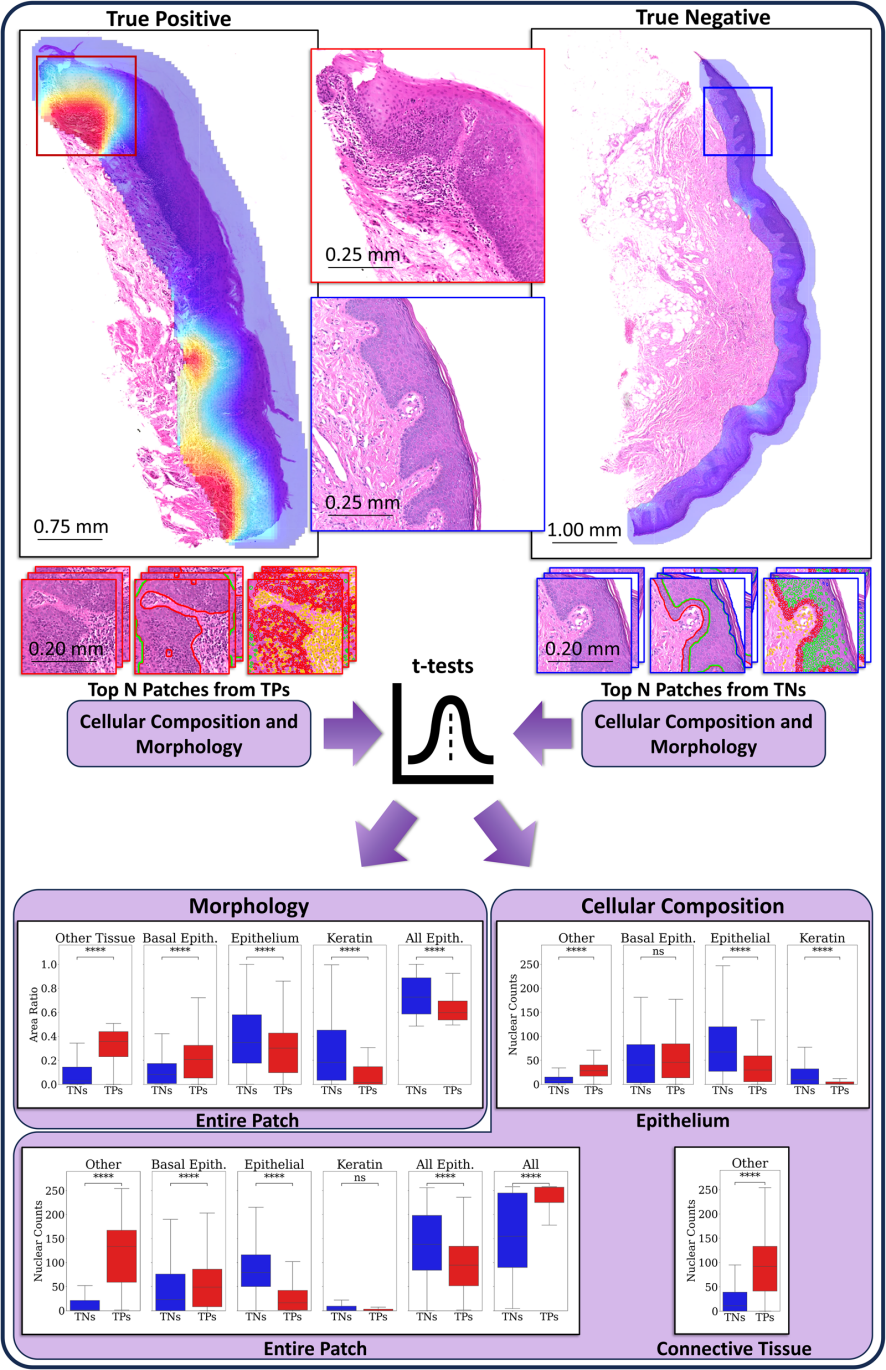
Feature analysis pipeline and results. An example mild OED case with our algorithm prediction heatmap overlaid (left), where our algorithm correctly predicted the case to transform to malignancy. On the right is an example mild case that our algorithm correctly predicted would not transform. The diagram shows how the top predicted patches from true positive (TP) cases (left), and the top predicted patches from the true negative (TN) cases (right), are taken and morphology and cellular composition features are found (based on the HoVer-Net+ segmentations). This was performed over the entire Sheffield cohort and t-tests (with FDR correction) were used to determine any differences. The bottom of the image has boxplots showing the distribution of nuclear counts (cellular composition) within the entire patch, the epithelium alone, and the connective tissue alone, of the top five predicted patches from true positive (TP) cases, and the top five patches from true negatives (TNs). We additionally give boxplots showing the distribution of area ratios (morphology) within the top five predicted patches from TPs, and the top five patches from TNs.

**Fig. 4:**
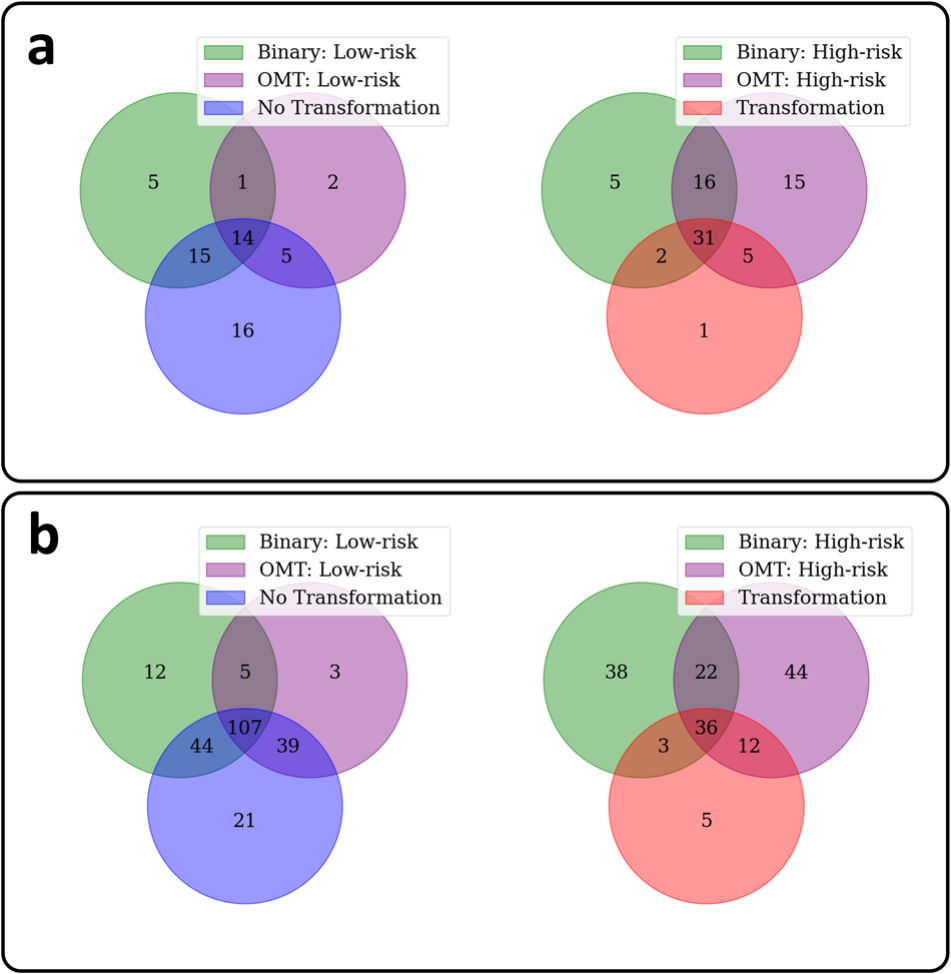
Venn diagrams comparing model predictions to the Binary grading system. **a** The produced Venn diagrams show the overlap in predictions between the *OMTscore* and binary grading system on an internal and **b** external validation.

In this section, we show the performance of our model, trained with patch-level morphological/spatial features, both quantitively, when compared to the pathologist grades (see Table 2) and qualitatively (see Fig. 3 for heatmaps, and Fig. 4 for Venn diagrams). On internal validation, our model attained competitive results with an AUROC of 0.77, outperforming both the WHO grade (AUROC = 0.68) and the binary grade (AUROC = 0.71). In total, our *OMTscore* had 48 true positives (TPs), 148 true negatives (TNs), 65 false positives (FPs), and 9 false negatives (FNs). In contrast, the binary grading system resulted in 40 TPs, 152 TNs, 61 FPs, and 17 FNs. For external validation on the Birmingham-Belfast cohort (see Table 3), our model achieved superior results in terms of AUROC and recall (AUROC = 0.75, Recall = 0.92) compared to both the WHO and binary grades. Our *OMTscore* had a total of 37 TPs, 20 TNs, 29 FPs, and 3 FNs, whilst the binary grading system had 34 TPs, 29 TNs, 20 FPs, and 6 FNs. The ROC curves for our proposed model are shown in Fig. 2a.

**Table 2:** Slide-level mean (standard deviation) results for transformation prediction on internal validation

	Sheffield (*n =* 270)			
Model	F1-score	Recall	Fall-out	AUROC
**OMTscore**	**0.57 (0.08)**	**0.84 (0.07)**	0.30 (0.12)	**0.77 (0.08)**
Binary Grade	0.51 (0.08)	0.70 (0.09)	0.28 (0.07)	0.71 (0.06)
WHO Grade G1	0.46 (0.08)	0.94 (0.07)	0.59 (0.07)	0.68 (0.05)
WHO Grade G2	0.34 (0.16)	0.41 (0.19)	**0.24 (0.08)**	0.58 (0.11)

WHO Grade G1 is mild vs moderate/severe cases, whilst WHO Grade G2 is mild/moderate vs severe cases. Best model/scores are given in bold.

**Table 3:** Slide-level mean (standard deviation) results for transformation prediction on external validation

	Birmingham (*n* = 47)				Belfast (*n* = 42)				Combined (*n* = 89)			
Model	F1-score	Recall	Fall-out	AUROC	F1-score	Recall	Fall-out	AUROC	F1-score	Recall	Fall-out	AUROC
**OMTscore**	0.44 (0.01)	0.87 (0.06)	0.57 (0.07)	0.73 (0.01)	**0.84 (0.02)**	**0.93 (0.03)**	0.69 (0.05)	**0.71 (0.03)**	0.69 (0.01)	**0.92 (0.04)**	0.60 (0.06)	**0.75 (0.01)**
Binary Grade	0.55	0.80	0.30	0.75	0.80	0.87	0.75	0.56	**0.72**	0.85	0.41	0.72
WHO Grade G1	**0.55**	**0.90**	0.38	**0.76**	0.79	0.87	0.83	0.52	0.71	0.88	0.49	0.69
WHO Grade G2	0.40	0.30	**0.05**	0.63	0.39	0.27	**0.25**	0.51	0.39	0.28	**0.10**	0.69

WHO Grade G1 is mild vs moderate/severe cases, whilst WHO Grade G2 is mild/moderate vs. severe cases. Best model/scores are given in bold.

The heatmaps produced by our model were inspected by a pathologist (SAK). They revealed prognostic areas with obvious or high grades of dysplasia, and a significant presence of immune cells within and around the epithelium. An example heatmap of a mild OED case is shown in Fig. 3 (top left), which was correctly predicted by our model to transform. Further examination of the hotspots indicated a focus on dysplastic areas with a prominent lymphocytic infiltrate within the epithelium and peri-epithelial lymphocytes. We also provide Venn diagrams showing the overlap of binary grade and *OMTscore* patient stratifications on internal and external validation in Fig. 4. It is clear that both our pipeline and binary grades are frequently predicting the same slides as high-risk, having a high overlap, but with the *OMTscore* being more sensitive than the binary grade.

### Survival analyses

We further conducted survival analyses to determine the prognostic utility of our *OMTscore* when compared to the pathologist-assigned grading systems. See Fig. 2b for the Kaplan-Meier (KM) curves for the *OMTscore* and binary/WHO grades on the internal cohort. The *OMTscore* demonstrated a clear separation between the low- and high-risk cases, with strong concordance, (C-index = 0.74, *p* < 0.001) outperforming the binary grade (C-index = 0.69, *p* < 0.001) and WHO grade (C-index = 0.69). Results from the Cox proportional hazard model (see Table 4) showed that both the *OMTscore* (*p* < 0.001, HR = 8.48 [3.06, 21.30]) and binary grade (*p* < 0.005, HR = 3.96 [1.45, 11.10]) were statistically significant. The WHO grade (*p* = 1.00, HR = 1.06 [0.57, 2.04]) was not significant. The *OMTscore* exhibited the highest hazard ratio (HR), indicating better prognostic utility. No other clinical variables were found to be significant.

**Table 4:** Multivariate Cox Proportional Hazard Model output for malignant transformation based on the OMTscore and other clinical variables

	Internal Validation – Sheffield (*n* = 270)				External Validation – Combined (*n* = 89)			
	*p*	HR	Lower 95% HR	Upper 95% HR	*p*	HR	Lower 95% HR	Upper 95% HR
**OMTscore**	**<0.001**	**8.48**	**3.87**	**21.30**	0.32	3.01	0.71	20.62
**Binary Grade**	**<0.001**	**3.96**	**1.45**	**11.10**	0.14	2.64	0.70	8.84
WHO Grade	1.00	1.06	0.57	2.04	0.96	1.27	0.64	2.50
Age	0.54	1.01	0.98	1.03	1.00	1.00	0.97	1.02
Sex	0.60	1.34	0.71	2.51	0.81	1.29	0.61	2.62
Site	0.36	1.19	0.85	1.67	0.07	1.59	1.03	2.55

Best model/scores are given in bold.

For external validation, KM survival curves were presented for the Birmingham-Belfast cohort (Fig. 2b). The *OMTscore* exhibited statistically significant differences in KM curves (*p* = 0.02) according to a log-rank test. The *OMTscore* also achieved a comparable C-index of 0.60 compared to the WHO grade’s C-index of 0.64 (*p* = 0.003) and the binary grade of 0.65 (*p* < 0.001). Results from the multivariate Cox PH models (see Table 4) showed no variables to be statistically significant. However, both the binary grade (*p* = 0.14, HR = 2.64 [0.70, 8.83]), and *OMTscore* (*p* = 0.32, HR = 3.01 [0.71, 20.62]), had high hazard ratios, highlighting their prognostic utility over the other clinical variables.

### Feature analyses

In order to determine the most important features used by the model for predicting malignant transformation, we performed several analyses. First, we compare the cellular composition and morphology of the most predictive patches in correctly predicted cases. Second, we looked at the feature importance for the 168 morphological/spatial features, based on a Random Forest classifier (see Supplementary Material pp 5). Third, we study partial dependency probability plots (PDPs), to determine the effect each feature has on the predicted outcome in isolation. Together, these analyses give more explainability to the models predictions.

We analysed the most important features used by our model, in terms of cellular composition and morphology, by comparing the top five predictive patches in true positive cases to the top predicted patches in true negative cases on both internal and external validation cases (see Supplementary Fig. 4 for a random selection of patches and Supplementary Material pp 5 for the internal validation results). On external validation, patch-level nuclear counts revealed higher cellularity in true positive (TP) patches compared to true negatives (TNs) (Cohen’s *d* = 0.35, *p* < 0.001; see Fig. 3, Cellular Composition: Entire Patch), primarily driven by “other” nuclei in TPs (*d* = 1.30, *p* < 0.001). In contrast, there were more epithelial cells in TNs (labelled as “All Epith” in Fig. 3; *d* = 0.78, *p* < 0.001). When focussing on the nuclear counts within the epithelial region of the patch alone (Fig. 3, Cellular Composition: Epithelium), significant differences were found in the number of “other” nuclei within the epithelium (*d* = 1.16, *p* < 0.001). Additionally, there were more epithelial nuclei within the epithelial layer in TNs (*d* = 0.91, *p* < 0.001), while slightly more (but not significantly) basal epithelial nuclei were observed in TPs (*d* = 0.11, *p* = 0.13). A significant difference was also found in the number of keratin nuclei between classes (*d* = 0.47, *p* < 0.001). Lastly, Fig. 3 (Cellular Composition: Connective Tissue) illustrates a larger number of “other” nuclei within the connective tissue of TPs compared to TNs (*d* = 1.00, *p* < 0.001).

When analysing the distributions of tissue types (or morphology) within patches (Fig. 3, Morphology: Entire Patch), we found that TP patches had a higher ratio of connective tissue (presumed from “other” tissue) compared to TNs (*d* = 1.66, *p* < 0.001). This is consistent with the prior nuclear analysis showing more “other” nuclei in TP patches. Additionally, TP patches often had more basal tissue (*d* = 0.72, *p* < 0.001), but less epithelial tissue (*d* = 0.45, *p* < 0.001), compared to TNs. Interestingly, TNs had significantly more surface keratin compared to TPs (*d* = 0.52, *p* < 0.001). TN patches primarily contained the epithelium, whereas TP patches specifically were restricted to the basal layer and connective tissue.

**Fig. 5:**
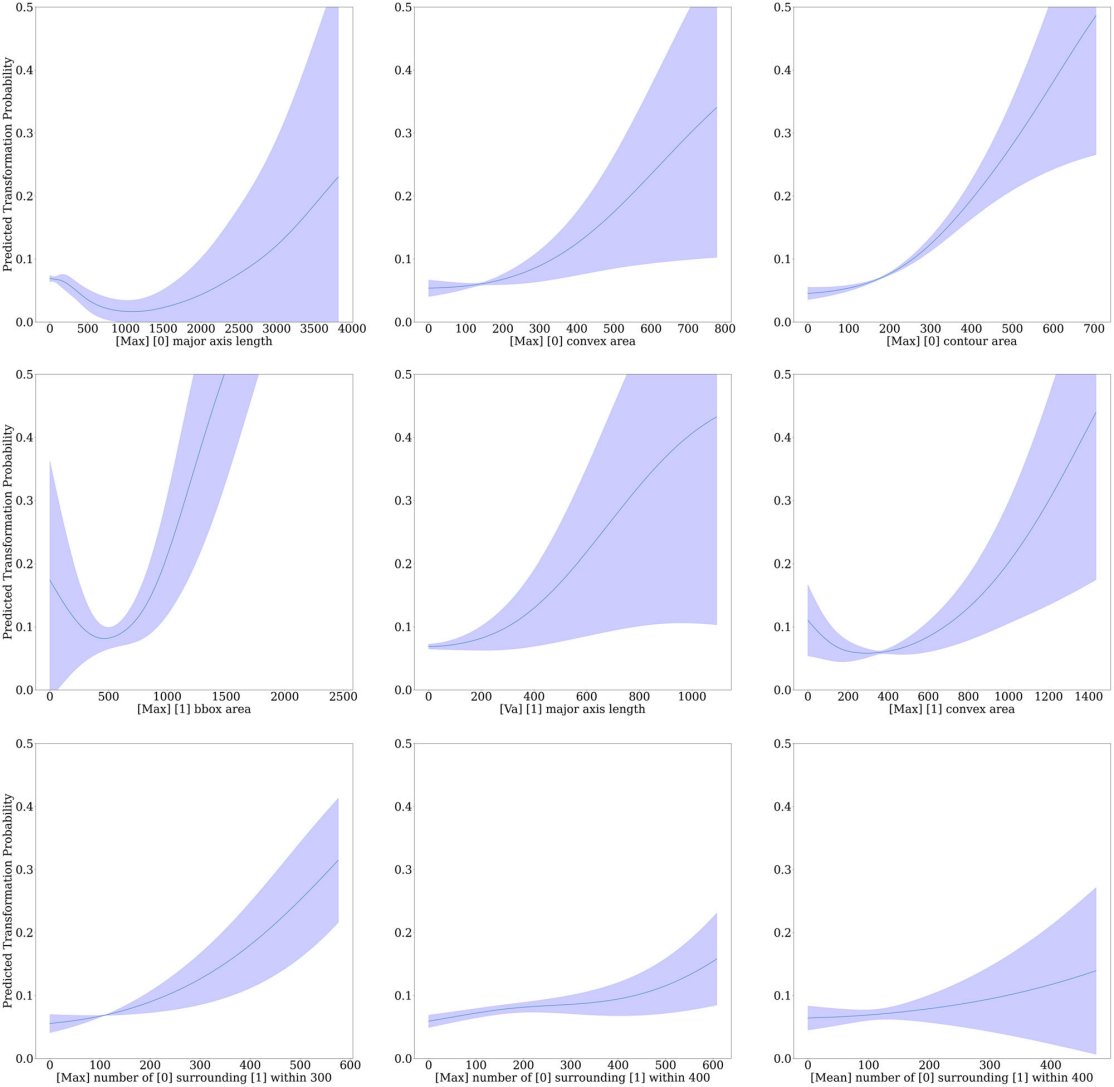
Partial dependency plots for the *OMTscore* on external validation. Partial Dependency Probability Plots are given for the *OMTscore* based on the entire external validation data. Here, “epithelial” nuclei are labelled as [1] and “other” nuclei are labelled as [0]. Distances are given in microns. The confidence intervals are based on the standard deviation across the three repeats of external validation experiments.

We produced PDPs for all features based on the entire external test set for the MLP model producing the *OMTscore*. PDPs give an indication of the importance of each individual feature in predicting transformation, with positive gradients giving a positive association. We give nine of the features that appeared to have the largest gradients in Fig. 5. Within the top row, these plots show clear positive associations between larger maximum major axis lengths, convex and contour areas in “other” nuclei, and malignant transformation. We see in the middle row the positive relationship between the maximum area (bounding box and convex area) of epithelial nuclei and variance in major axis length in epithelial nuclei, and malignant transformation. Finally, in the bottom row, we see a positive correlation between higher amounts of “other” nuclei surrounding epithelial nuclei. Results for internal validation can be seen in the Supplementary Material (pp 6).

## Discussion

We introduced the *OMTscore*, a risk score that incorporates interpretable morphological and spatial features for predicting OED transformation. Our approach involved first introducing a new model for simultaneous segmentation of intra-epithelial layers and nuclei in H&E WSIs. We then generated patch-level morphological/spatial features, resembling cytological features used by pathologists for OED grading (e.g. anisonucleosis, nuclear pleomorphism). These features were fed into a shallow neural network, yielding high predictive performance for OED transformation.

Overall, our *OMTscore* achieved comparable performance to pathologist-assigned grades on external validation. Notably, the *OMTscore* attained a higher AUROC and sensitivity than the binary grading system, although this came with a higher false alarm rate. In contrast, the WHO and binary grades gained slightly higher C-indexes. Despite this, our *OMTscore* still effectively distinguished between low- and high-risk cases. In relation to the multivariate Cox models, both the *OMTscore* and binary grade demonstrated prognostic significance on internal validation. However, external validation did not identify any statistically significant variables, likely influenced by the lower sample size. Nevertheless, both the binary grade and *OMTscore* had high hazard ratios, underlining their potential as strong prognostic indicators. These findings highlight the prognostic utility of our *OMTscore*, with its enhanced sensitivity aiding in the early detection of high-risk lesions. This has important implications for patient care, potentially enabling more timely interventions and reducing the risk of cancer development.

Our model generalised well to new domains on external validation, but despite this, there was a drop in performance. We offer two explanations for this. First, the external dataset exhibited drastically lower survival rates (see Supplementary Fig. 10) compared to the Sheffield dataset, reflecting the clinical reality and underscoring the complexity of the problem. Second, we suggest that this drop may be partially attributed to HoVer-Net+’s limited generalisability to unseen domains. Visual inspection revealed unsatisfactory segmentations in a small subset of cases within the external cohort, which, when excluded, led to improved model performance in terms of AUROC (see Supplementary Material pp 6). This suggests that the performance decrease may not solely reflect the quality of the proposed transformation prediction pipeline, but rather indicate a need for further enhancing HoVer-Net + ’s generalisability.

We additionally acknowledge the variations in *OMTscore*’s performance across external cohorts. While our model outperforms grading systems on the Belfast cohort, it shows comparable AUROC but lower F1-scores on the Birmingham cohort. This has also resulted in our model achieving substantially higher AUROC scores on the Belfast cohort than the Birmingham cohort, but much lower F1-scores. This disparity can be attributed to our model’s high sensitivity, combined with the Birmingham cohort having fewer cases that transformed to malignancy (*n* = 10) when compared to Belfast (*n* = 30). This variation in the number of events is a clear indication of a type II prior (domain) shift between external cohorts^37^, and is the clinical reality of retrospective data. However, these individual cohorts are relatively small, and so we argue that evaluating our model (and grading systems) across both cohorts combined provides a more comprehensive understanding. We also add that when we performed an additional quality control step to find poorly segmented cases (see the Supplementary Material pp 6), all were in the Birmingham cohort. This further supports our hypothesis that HoVer-Net+ may not generalise as effectively to external data, contributing to the drop in prediction performance. Overall, our model achieved higher AUROC and recall across both cohorts combined, demonstrating strong prognostic utility.

Our model’s utilization of domain-agnostic morphological/spatial features contributed to its generally robust performance on external cohorts, whilst enhancing interpretability. Thus, the inclusion of PDPs and Random Forest analyses provided valuable insights into the behaviour of morphological and spatial features during external validation. While PDPs revealed consistent patterns between internal (see Supplementary Material pp 6) and external validation, RF analyses (see Supplementary Material pp 5) unveiled differences in feature importance. Notably, spatial features were found to be most prominent in internal validation, whereas a combination of morphological/spatial features proved most important in external validation. This discrepancy prompts a critical examination of nuclear classification robustness on the external test set. Within Supplementary Fig. 3, we show that nuclear classification is visibly poorer in some cases on external validation. Thus, the potential reliance on spatial features for discriminating between nuclear types may have contributed to less accurate signals for the model, possibly influencing the observed performance drop. This therefore again suggests that future work should aim to improve the generalisability of HoVer-Net + .

The feature analyses revealed that non-transforming cases exhibit more epithelial cells, while transforming cases exhibited higher counts of *basal* epithelial cells, and “other” nuclei in both connective tissue and the epithelium. The PDP analyses further supported this, indicating that the density of “other” nuclei surrounding epithelial nuclei was positively associated with malignant transformation. These findings are consistent with previous studies noting increased immune cell infiltration in oral lesions progressing to OSCC^38^, and recognising distinct immune-related subtypes in moderate/severe OED^39^. Given that the epithelium typically only contains epithelial nuclei or intra-epithelial lymphocytes (IELs), we suggest that these “other” nuclei within the epithelium are IELs. We additionally posit that the elevated density of “other” nuclei in the connective tissue likely represents peri-epithelial lymphocytes (PELs). This is further supported by the work of Bashir et al.^36^, who showed a higher density of PELs in cases that exhibited malignant transformation. Together, these results suggest that an increased density of IELs and PELs may signify a predisposition for the lesion to progress to cancer. This is intriguing, given that in oral cancer, a higher density of immune cells typically signifies a robust immune response and better outcomes. This finding calls for thorough exploration, emphasizing the crucial need to understand immune mechanisms in OED and identify specific cell profiles linked to malignant transformation. Such insights may facilitate the development of more targeted treatments, including exploring the potential role of immunotherapy in managing high-risk lesions. This approach holds particular promise for patients unsuitable for surgery, emphasizing the significance of advancing our understanding of immune dynamics in OED for improved therapeutic interventions.

While our study represents one of the first endeavours to predict OED malignant transformation, which has been validated on multiple external cohorts, it carries several limitations. This may be the largest known OED dataset with clinical outcomes for computational pathology, yet the sample size is still relatively small, with the training data sourced from a single centre (albeit using two scanners), and annotated by a sole pathologist. Additionally, the retrospective nature of our study poses inherent constraints. Future research should therefore expand on both the methods and findings of this work, whilst evaluating the utility of the *OMTscore* on an internationally acquired, multi-centric, and prospectively collected dataset, with multiple independent annotators, to ensure unbiased testing. Furthermore, exploring additional pathologist-derived patch-level cytological features, such as hyperchromatism and mitoses, could enhance the predictive capability of our model. In the Supplementary Material (pp 6), we provide insights into the potential importance of monitoring mitosis using published detection methods^40,41^. Architectural changes, such as irregular epithelial stratification and drop-shaped rete pegs, also warrant further exploration^35^. Finally, our feature analysis mainly focussed on true positive and true negative patches, driven by our goal to evaluate the model’s performance in correctly identifying transforming cases. However, we suggest future work should also examine false positives and false negatives to identify features contributing to incorrect model predictions and guide further optimization efforts.

In summary, our study has introduced an automated pipeline for predicting OED transformation using a state-of-the-art deep learning framework and patch-level morphological/spatial features. Our results demonstrate the strong prognostic significance and generalisability of our model compared to manual grades on internal and external cohorts. This has significant clinical implications for patient management, offering a potentially more accurate and objective prediction method. Our study paves the way for future research and the potential to enhance patient outcomes through early detection and intervention. However, further investigations are required to identify additional slide-level features and validate the model on larger external cohorts with longer follow-up periods.

## Methods

### Study data

The study cohort used for training our models consisted of subjects collected retrospectively between 2008 and 2016 from the Oral and Maxillofacial Pathology archive at the School of Clinical Dentistry, University of Sheffield, UK. Sections were newly cut (4 µm thickness) and H&E stained from formalin-fixed paraffin embedded blocks.

In total, 244 cases were assessed for eligibility. This comprised of 321 slides with a histological diagnosis of OED, scanned using either a Hamamatsu NanoZoomer 360 (Hamamatsu Photonics, Japan) or an Aperio CS2 (Leica Biosystems, Germany) digital slide scanner at 40× objective power (0.2258 mpp and 0.2520 mpp, respectively) to obtain digital WSIs. Of these 244 cases, only 202 cases met the study inclusion criteria (279 slides; see the Supplementary Materials pp 2 for inclusion criteria). Further, clinical information including patient age, sex, intraoral site, OED grade (binary and WHO 2017), and transformation status, was available for just 193 cases (270 slides). The case transformation information was gathered from multiple sources, primarily patient clinical systems. The evaluation involved a thorough assessment of patient records and the diagnostic database, which included both electronic and physical files (by a clinician, HM). Specifically, transformation was defined as the progression of a dysplastic lesion to OSCC at the same clinical site within the follow-up period. Multiple certified/consultant pathologists independently evaluated the cases when initially reported using the WHO grading system (PMS, PMF, DJB, KDH), to ensure diagnostic consistency. Blind re-evaluation was performed by an Oral & Maxillofacial Pathologist (SAK) and an Oral Surgeon specialising in OED analysis (HM), to confirm the WHO (2017) grade and assign binary grades. In total, the cohort included 193 unique OED patients (270 slides) with 42 patients (57 slides) exhibiting malignant transformation. Slides from the same patients were consistently assigned to the same fold during training/internal cross-validation. A summary of the cohort is provided in Supplementary Table 1, and a CONSORT diagram is also given in Supplementary Fig. 1.

For training our segmentation models, one expert pathologist (SAK) exhaustively manually delineated the intra-epithelial layers (basal, epithelial, and superior keratin layers) in 59 OED cases, in addition to nine controls (collected with the Aperio CS2 scanner as per the above protocols), using our in-house WASABI software (a customised version of HistomicsTK^42^). We then generated tissue masks for each of the segmented WSIs via Otsu thresholding and the removal of small objects and holes in the segmentation mask. A layer mask was then generated for each WSI by combining the layer segmentations with the tissue mask.

The manual segmentation of individual nuclei within WSIs is laborious and subject to inter/intra-rater variability. Thus, nuclear instance masks were generated for a small subset of cases, 30 regions of interest (one ROI per case), where a pathologist (SAK) annotated each nucleus as either epithelial or “other”. The point annotations were used within the NuClick framework to generate nuclear boundaries^21^. NuClick is a deep learning framework that takes a raw image and a guiding signal “click” as an input and then produces a nuclear instance boundary as an output. This method has been found to be superior to fully automated approaches for generating nuclear instance segmentations, particularly in the cases of touching/overlapping nuclei^21^. To ensure that all nuclear segmentations were of a high quality, the masks were then manually refined when found to be visibly incorrect. A total of 71,757 labelled nuclei segmentations were obtained across the 30 ROIs, which were used to train our segmentation models.

For external validation, OED cases from two independent centres, Birmingham and Belfast, were recruited. A total of 47 OED patients’ data were collected from Belfast and 71 OED cases were collected from Birmingham. The Birmingham and Belfast slides were scanned at 40× objective power using a Pannoramic 250 (3DHISTECH Ltd., Hungary; 0.1394 mpp) and an Aperio AT2 (Leica Biosystems, Germany; 0.2529 mpp) scanner, respectively. On receipt of cases, all cases were blindly re-evaluated by SAK to confirm histological grade (WHO 2017 and binary) and ensure the inclusion criteria were met. They additionally had time to transformation data. The combined Birmingham-Belfast external validation cohort consisted of 118 unique OED cases, however, of these cases, 29 did not meet the study criteria. This resulted in 89 OED cases (89 slides), with 40 cases transitioning to malignancy. A summary of this cohort is provided in Supplementary Table 1, and a CONSORT diagram is also given (see Supplementary Fig. 1).

### Analytical workflow overview

To predict the OED risk score, we implemented a multi-step pipeline (see Fig. 1). First, a deep learning model was trained to automatically segment the epithelium and nuclei. This model was then used for inference on all slides. For the downstream analysis, the slides were tessellated into smaller tiles, and tile-level features were generated based on the nuclear segmentations (in tiles with ≥50% epithelium). These features were used to train a shallow neural network for slide-level prediction. The algorithm was internally validated on the Sheffield cohort, and subsequently validated on the external cohort, consisting of cases from two independent centres.

### Layer and nuclear segmentation

To generate layer and nuclear segmentation for each WSI in our cohort, we trained/tested HoVer-Net+ on the internal Sheffield cohort, using the ground-truth annotations. HoVer-Net+ is an encoder-decoder-based CNN that simultaneously segments and classifies nuclear instances, and semantically segments the epithelial layers^20^. We used this model to semantically segment the intra-epithelial layers (e.g. basal, epithelial, and keratin) and other tissue (e.g. connective tissue), whilst also segmenting and classifying nuclear instances as epithelial or “other” nuclei. Here, “other” nuclei are any form of nuclei that are not epithelial nuclei, (i.e. connective/inflammatory). We trained HoVer-Net+ using a multi-stage approach, based on the layer segmentations of 56 cases/controls and the nuclear segmentation masks of 24 cases/controls. The model was then tested on the layer segmentation of 12 cases/controls and the nuclear segmentations of 6 cases/controls. HoVer-Net+ takes 256×256 patches at 20× magnification (0.50 mpp), and produces nuclear instance segmentation/classification maps, and semantic segmentations of the epithelial layers. Note, that a small patch size of 256 (at 20×) is necessary for accurate nuclear segmentation. The training involved two phases, with the decoder branches trained for 20 epochs in phase one, and all branches trained for 30 epochs in phase two. A batch size of 8 and 4 on each GPU was used across these phases, respectively. The Adam optimiser was used with a learning rate that decayed initially from 10^-4^ to 10^-5^ after 10 epochs in each phase. Data augmentations such as flip, rotation, blur, and colour perturbation were applied during training. We also tested the effect of stain augmentation using the TIAToolbox^34^ implementation of the Macenko method^43^ that has been shown to effectively counter scanner-induced domain-shifts to make our model more generalisable^40,44^. For detailed information on model training, please refer to the Supplementary Material (pp 3-4). Following model training, we used HoVer-Net+ for inference on all slides from both the internal and external cohorts.

### Slide-level transformation prediction

After segmentation, each WSI was tessellated into smaller 512 × 512 tiles (20× magnification, 0.50 mpp) with 50% overlap. We used this tile size to ensure that each tile contained enough information for the prediction task, in line with previous studies^29,36^. We then generated tile-level features for use in a weakly supervised model for transformation prediction. For each tile, we calculated 104 morphological and 64 spatial features. The morphological features were obtained from 13 shape features for each nucleus in a tile (eccentricity, convex area, contour area, extent, perimeter, solidity, orientation, radius, major/minor axis, equivalent diameter, bounding box area/aspect ratio) with four tile-level statistics (mean, minimum, maximum, standard deviation) per nuclear type (epithelial and other). This resulted in 104 morphological features per tile. We computed the number of different nuclear types within a small radius of a nuclear instance, resulting in four counts per tile (number of epithelial nuclei around another nucleus, number of epithelial nuclei around epithelial nuclei, number of other nuclei around epithelial nuclei, and finally the number of other nuclei around other nuclei) over four varying radii (100, 200, 300 and 400 pixel radii). Finally, we took tile-level summary statistics (mean, minimum, maximum, standard deviation) across these 16 features, resulting in 64 spatial features per tile. We chose to use these 168 morphological/spatial features in preference to “deep” features output by CNNs, such as in traditional prediction tasks^25,29,31,33^, to offer transparency and explainability to the model inputs.

For slide-level prediction, a MLP was trained using the iterative draw-and-rank (IDaRS) method introduced by Bilal et al.^29^ leveraging our tile-level features. The output of our MLP is referred to as the *OMTscore*. The MLP architecture consisted of three layers with 168 nodes in the input layer, 64 nodes in the hidden layer, and 2 nodes in the output layer. We employed a leaky ReLU activation function and dropout (0.2) after the hidden layer. The MLP models were trained with a symmetric cross-entropy loss function and the Adam optimiser. This loss function was chosen as it has been shown previously to help overcome errors associated with weak labels^29,45^. IDaRS sampling was performed with parameter values of *k* = 5 for the top predictive patches and *r* = 45 random patches, using a batch size of 256. The models underwent training for 100 epochs and were evaluated through five-fold cross-validation (repeated 3 times, with random seeds) for internal validation. To generate slide-level predictions we calculated the average probability over each tile in a slide to predict transformation. This method demonstrated optimal performance during internal cross-validation. A threshold was determined based on the internal cross-validation and applied to external validation. External validation involved combining the entire Sheffield cohort as a discovery cohort for model training, with validation performed on the combined Birmingham-Belfast cohort (repeated 3 times, with random seeds). It’s important to note that the use of the IDaRS sampling method ensures robust predictions. By drawing from both random and informative (from the previous iteration) tiles, the model is trained to achieve discrimination between different tiles, even in the presence of imbalanced data. This methodology aims to prevent slide-level predictions from being hindered by small numbers of positive tiles.

To determine the utility of our predicted *OMTscore*, we compared its prognostic capability against both the pathologist-assigned WHO and binary grading systems. Whilst we note that these systems do not aim to directly predict cases that will transform to malignancy; we argue that the goal of the grading systems is to give patient prognosis and stratification, in order to inform treatment decisions. This is ultimately what we are aiming to do with the *OMTscore*, thus making a fair comparison.

### Survival analyses

Survival analyses were conducted to assess the prognostic significance of the *OMTscore*, and the manually-assigned WHO/binary grades, in predicting transformation-free survival. The *OMTscore* indicated whether the algorithm predicted the case to transform (high-risk) or not (low-risk). Kaplan-Meier curves were generated using the Python *lifelines* package, and log-rank tests were used to determine the statistical significance of the grade stratification (for OMT, WHO, and binary grades). Additionally, a multivariate Cox proportional hazards model was employed, incorporating sex, age, lesion site, binary, and WHO grade, to predict transformation-free survival. The purpose of this analysis was to validate the prognostic significance of the predicted *OMTscore* relative to other clinical variables. This analysis was performed on both the internal and external cohorts. Transformations were right-censored at eight years across these analyses to ensure consistency between internal and external cohorts.

### Feature analyses

We performed several post-hoc analyses based on both our internal and external validation cohorts to add a level of explainability to our model predictions. First, we focused on the nuclear count features within the top five predicted patches of correctly predicted positive slides (true positives) and compared them to the top five predicted patches of correctly predicted negative slides (true negatives) within the testing subsets. Two-tailed t-tests were performed with multiple comparison correction (false discovery rate, FDR) to determine the statistical significance of any observed differences. We conducted three comparative analyses of the cellular composition of the top predicted patches: (1) nuclei within the entire patch (other, basal, epithelial, keratin), 2) nuclei within the epithelium (other, basal, epithelial, keratin), and 3) nuclei within the connective tissue surrounding the patch (e.g., peri-epithelial “other” nuclei). In addition, we analysed the tissue type ratios (morphology) within these top-predicted patches. Note, as multiple runs of the experiments were conducted, these analyses contains true positives and true negatives from correctly predicted cases from all runs. These experiments enabled us to determine any associations between nuclear types/areas and the predicted outcome.

Second, we investigated which of the 168 morphological/spatial features used to train our MLP were most important for making the final prediction. We achieved this by training a Random Forest classifier using the top five correctly predicted patches per correctly predicted case by our MLP model, utilizing the 168 nuclear features. Subsequently, we ranked the feature importance, measured by mean decrease in impurity (MDI), and identified the top ten features. To ascertain their statistical significance, we conducted two-tailed t-tests with FDR correction.

Third, we also explored the PDPs for our MLP model when tested on both internal and external cases. We systematically adjusted the value of each of the 168 input features, one at a time, from its minimum to its maximum value in 100 increments, and plotted this against the model’s predicted probability output across all cases. These analyses provide insights into the significance of each individual feature in predicting transformation.

### Evaluation metrics

We evaluated the layer segmentation using the F1-score aggregated over all image patches. For nuclear instance segmentation, we assessed the Panoptic Quality (PQ), detection quality (DQ, or F1-score), and segmentation quality (SQ). Additionally, we report the Dice score comparing segmented nuclei against the background, and the aggregated Jaccard Index (AJI). We also calculate the average values over all images for: F1-score for detection (F_d_, all nuclear types) and F1-score for classification (F_c_) for each nucleus type (e.g. F_c_^b^ for basal epithelial nuclei, F_c_^e^ for epithelial nuclei, and F_c_^o^ for other nuclei). Detailed descriptions of these metrics can be found in Graham et al.^19^. When evaluating the model’s performance in predicting transformation, we calculated the average F1-score and AUROC across all slides. The F1-score is the harmonic mean of recall (sensitivity) and precision, and thus provides a balance between false positives and false negatives. In addition, we also include the model recall (sensitivity) and fall-out (false positive rate).

### Reporting summary

Further information on research design is available in the Nature Research Reporting Summary linked to this article.

### Supplementary information

Reporting Summary

Supplementary Material

## Data Availability

All the data derived from this study are included in the manuscript. We are unable to share the whole slide images and clinical data, due to restrictions in the ethics applications.

## Code availability

We have made the model inference code publicly available online, along with model weights (adamshephard/OMTscoring_inference(github.com)). All code was written with Python 3.10 and PyTorch 2.0, along with TIAToolbox 1.4.0.
