# Supplementary material for: A fully automated and explainable algorithm for predicting malignant transformation in oral epithelial dysplasia
Source: NPJ Precis Oncol. 2024 Jun 28;8:137. doi: 10.1038/s41698-024-00624-8 (PMC11213925; doi:10.1038/s41698-024-00624-8)
Supplement: Supplementary file 1 — Reporting Summary [file 41698_2024_624_MOESM1_ESM.pdf]

nature portfolio

Corresponding author(s): Prof. Nasir Rajpoot

Last updated by author(s): Jan 3, 2024

Reporting Summary

Nature Portfolio wishes to improve the reproducibility of the work that we publish. This form provides structure for consistency and transparency in reporting. For further information on Nature Portfolio policies, see our Editorial Policies and the Editorial Policy Checklist.

Statistics

For all statistical analyses, confirm that the following items are present in the figure legend, table legend, main text, or Methods section.

n/a	Confirmed	
☐	☒	The exact sample size (*n*) for each experimental group/condition, given as a discrete number and unit of measurement
☐	☒	A statement on whether measurements were taken from distinct samples or whether the same sample was measured repeatedly
☐	☒	The statistical test(s) used AND whether they are one- or two-sided<br>*Only common tests should be described solely by name; describe more complex techniques in the Methods section.*
☐	☒	A description of all covariates tested
☐	☒	A description of any assumptions or corrections, such as tests of normality and adjustment for multiple comparisons
☐	☒	A full description of the statistical parameters including central tendency (e.g. means) or other basic estimates (e.g. regression coefficient) AND variation (e.g. standard deviation) or associated estimates of uncertainty (e.g. confidence intervals)
☐	☒	For null hypothesis testing, the test statistic (e.g. *F*, *t*, *r*) with confidence intervals, effect sizes, degrees of freedom and *P* value noted<br>*Give P values as exact values whenever suitable.*
☒	☐	For Bayesian analysis, information on the choice of priors and Markov chain Monte Carlo settings
☐	☒	For hierarchical and complex designs, identification of the appropriate level for tests and full reporting of outcomes
☐	☒	Estimates of effect sizes (e.g. Cohen's *d*, Pearson's *r*), indicating how they were calculated

Our web collection on statistics for biologists contains articles on many of the points above.

Software and code

Policy information about availability of computer code

Data collection	Collection of clinical metadata was performed using Microsoft Excel 365, whereas digitised whole-slide images were collected using Aperio CS2, Hamamatsu NanoZoomer 360, 3DHistech Pannoramic and Aperio AT2 scanners.
Data analysis	All image analysis was performed in Python 3.7.12. Analysis of the clinical data and survival analyses were performed using Lifelines 0.27.0.

For manuscripts utilizing custom algorithms or software that are central to the research but not yet described in published literature, software must be made available to editors and reviewers. We strongly encourage code deposition in a community repository (e.g. GitHub). See the Nature Portfolio guidelines for submitting code & software for further information.

Data

Policy information about availability of data

All manuscripts must include a data availability statement. This statement should provide the following information, where applicable:

- Accession codes, unique identifiers, or web links for publicly available datasets
- A description of any restrictions on data availability
- For clinical datasets or third party data, please ensure that the statement adheres to our policy

The code and model weights for reproducing the results is available at https://github.com/adamshephard/OMTscoring_inference. The histology image data is not available due to ethics restrictions.

Research involving human participants, their data, or biological material

Policy information about studies with human participants or human data. See also policy information about sex, gender (identity/presentation), and sexual orientation and race, ethnicity and racism.

Reporting on sex and gender	No data was collected on self reported gender.<br><br>Sex was tested as a covariate in the survival analyses, however, was not found to be significant.
Reporting on race, ethnicity, or other socially relevant groupings	No racial, ethical or socially relevant groupings were used.
Population characteristics	For the discovery cohort, the median age of the patients used was 64, with 57/270 slides exhibiting malignant transformation. For external, the median age was 61, with 40/89 cases having malignant transformation.
Recruitment	A purposive sampling method was used to acquire consecutive OED cases. Data collected was fully anonymised. Discovery and internal validation cohort included n = 193 patients collected between 2008 and 2016 from the Oral and Maxillofacial Pathology archive at the School of Clinical Dentistry, University of Sheffield, UK. External validation was performed on cohorts collected from two independent centres, Birmingham and Belfast. A total of 47 OED patients' data were collected from Belfast and 71 OED cases were collected from Birmingham.
Ethics oversight	Ethical approval for the study was obtained from the NHS Health Research Authority West Midlands (18/WM/0335).

Note that full information on the approval of the study protocol must also be provided in the manuscript.

Field-specific reporting

Please select the one below that is the best fit for your research. If you are not sure, read the appropriate sections before making your selection.

☒ Life sciences ☐ Behavioural & social sciences ☐ Ecological, evolutionary & environmental sciences

For a reference copy of the document with all sections, see nature.com/documents/nr-reporting-summary-flat.pdf

Life sciences study design

All studies must disclose on these points even when the disclosure is negative.

Sample size	In comparison to previously published works, the cohort for this study was sufficiently large (n = 193) with an additional external cohort (n = 89) for validation as well. Effective sample size i.e., number of events were n = 57 and n = 40 for malignant transformation, respectively. This is the largest, multi-centric OED dataset, with transformation information, to date.
Data exclusions	HPV-related OED and verrucous lesions were excluded. This was determined through morphological analysis, as these entities exhibit reportedly different features and behaviour. Cases were additionally excluded if there was insufficient availability of epithelial tissue (i.e. excluding tangentially cut sections, tissue with artefacts), the slide was of poor staining quality, or there was incomplete/irretrievable follow-up data.
Replication	The AI model trained on the internal discovery cohort was used to make predictions on external validation cohort and similar findings were replicated.
Randomization	For the discovery phase the participants were allocated following stratified random sampling to ensure sufficient number of events of interest in the sub strata. Similarly, a subset was separated for internal validation whereas an external cohort was used for independent validation.
Blinding	Blinded stratified random sampling was used for allocation.

Reporting for specific materials, systems and methods

We require information from authors about some types of materials, experimental systems and methods used in many studies. Here, indicate whether each material, system or method listed is relevant to your study. If you are not sure if a list item applies to your research, read the appropriate section before selecting a response.

Materials & experimental systems

n/a	Involved in the study
☒	☐ Antibodies
☒	☐ Eukaryotic cell lines
☒	☐ Palaeontology and archaeology
☒	☐ Animals and other organisms
☒	☐ Clinical data
☒	☐ Dual use research of concern
☒	☐ Plants

Methods

n/a	Involved in the study
☒	☐ ChIP-seq
☒	☐ Flow cytometry
☒	☐ MRI-based neuroimaging

Plants

Seed stocks	*Report on the source of all seed stocks or other plant material used. If applicable, state the seed stock centre and catalogue number. If plant specimens were collected from the field, describe the collection location, date and sampling procedures.*
Novel plant genotypes	*Describe the methods by which all novel plant genotypes were produced. This includes those generated by transgenic approaches, gene editing, chemical/radiation-based mutagenesis and hybridization. For transgenic lines, describe the transformation method, the number of independent lines analyzed and the generation upon which experiments were performed. For gene-edited lines, describe the editor used, the endogenous sequence targeted for editing, the targeting guide RNA sequence (if applicable) and how the editor was applied.*
Authentication	*Describe any authentication procedures for each seed stock used or novel genotype generated. Describe any experiments used to assess the effect of a mutation and, where applicable, how potential secondary effects (e.g. second site T-DNA insertions, mosiacism, off-target gene editing) were examined.*
