# Supplementary material for: A fully automated and explainable algorithm for predicting malignant transformation in oral epithelial dysplasia
Source: NPJ Precis Oncol. 2024 Jun 28;8:137. doi: 10.1038/s41698-024-00624-8 (PMC11213925; doi:10.1038/s41698-024-00624-8)

**Supplementary Materials**

Title: A Fully Automated and Explainable Algorithm for Predicting Malignant Transformation in Oral Epithelial Dysplasia

Authors: Adam J Shephard, Raja Muhammad Saad Bashir, Hanya Mahmood, Mostafa Jahanifar, Fayyaz Minhas, Shan E Ahmed Raza, Kris D. McCombe, Stephanie G. Craig, Jacqueline James, Jill Brooks, Paul Nankivell, Hisham Mehanna, Syed Ali Khurram, Nasir M Rajpoot

**Table of Contents**

[**Supplementary Methods** 2](#_Toc161220495)

[**Study Data** 2](#_Toc161220496)

[**Layer and Nuclear Segmentation** 2](#_Toc161220497)

[**Supplementary Results** 3](#_Toc161220498)

[**Layer and Nuclear Segmentation** 3](#_Toc161220499)

[**Added Value of HoVer-Net+** 4](#_Toc161220500)

[**Slide-level Transformation Prediction with our MLP** 5](#_Toc161220501)

[**External Validation Exclusion of Poor Segmentations with our MLP** 6](#_Toc161220502)

[**Comparison to ResNet34-IDaRS Method for Transformation Prediction** 6](#_Toc161220503)

[**Supplementary Discussion** 7](#_Toc161220504)

[**The Difficulty of the Task** 7](#_Toc161220505)

[**Domain Generalisation and Future Work** 7](#_Toc161220506)

[**Supplementary References** 9](#_Toc161220507)

[**Supplementary Tables** 10](#_Toc161220508)

[**Supplementary Figures** 14](#_Toc161220509)

**Supplementary Methods**

**Study Data**

WSIs were collected to the Sheffield cohort based on the following inclusion criteria: a histologically confirmed diagnosis of oral epithelial dysplasia (OED), of varying grades (mild, moderate and severe) and at least five years of follow-up data from the initial diagnosis (including transformation status). It was ensured that the sample included a sufficient number of cases that had transformed to malignancy. A purposive sampling method was used to acquire consecutive OED cases. These cases had been independently evaluated (between 2008-2016) by at least two certified/consultant pathologists (PMS, PMF, DJB, KDH) to provide an original diagnosis based on the WHO grading system. Prior to the inclusion of these cases in this study, each case was blindly re-evaluated by an Oral & Maxillofacial Pathologist (SAK) and an Oral Surgeon with a specialist interest and expertise in OED analysis (HM) to confirm histological grading using WHO (2017) and binary systems. Cases with disagreement were resolved through discussion within the team. Following this, HPV-related OED and verrucous lesions were excluded. This was determined through morphological analysis, as these entities exhibit reportedly different features and behaviour. Cases were additionally excluded if there was insufficient availability of epithelial tissue (i.e. excluding tangentially cut sections, tissue with artefacts), the slide was of poor staining quality, or there was incomplete/irretrievable follow-up data. This resulted in a total of 193 unique OED cases (with 270 slides) that are used in the analysis for the internal Sheffield cohort. Similarly for the external cohorts, on receipt of cases, all cases were blindly re-evaluated by SAK to confirm histological grade (WHO 2017 and binary) and ensure the inclusion criteria was met. The final external Birmingham-Belfast cohort consisted of 89 unique OED cases (with 89 slides). A summary of the internal/external cohorts are provided in Supplementary Table 1. We also provide a CONSORT diagram in Supplementary Figure 1 showing case collection.

**Layer and Nuclear Segmentation**

We aimed to make HoVer-Net+ as generalisable as possible; thus, in the training of our model we further split up our Sheffield dataset into smaller cohorts for testing the model generalisability. Thus of the 68 cases/controls that we had layer (and some nuclear) annotations for, we further split it up into two cohorts, cohort 1 (C1) and cohort 2 (C2; see Supplementary Table 2 for breakdown), so that each cohort had images from two different scanners.

HoVer-Net+ is multi-task learning method, thus, it is assumed that the training of the encoder via different datasets/tasks will only increase its generalisability and performance (3). To train our HoVer-Net+ model, we have taken a multi-stage, multi-cohort approach. At 20× magnification (0.50 mpp), it is logical that upon patch extraction, we will produce many more patches with layer segmentations (68 WSIs) than nuclear annotations (30 ROIs). Thus, the primary step of our training schemes will be to train the encoder and LS decoder for layer segmentation. Following this, we could then train the nuclear segmentation/classification decoders (NP/HoVer/NC branches). We tessellated our WSIs, layer masks and nuclear instance maps into smaller patches of size 256 × 256 (no overlap) at 20× magnification (0.50 mpp). For C1, this resulted in 1,139 patches with nuclear instance maps (and corresponding layer masks), and 24,532 patches with layer masks. For C2, this resulted in 866 patches with nuclear instance maps, and over 7,731 patches with layer masks. For training purposes, an 80/20 train/test split was used for all models. Our training scheme consists of three stages:

1. We first aim to train HoVer-Net+ for layer segmentation alone, based on all the layer segmentation patches (model: HoVer-Net+_LS_).
2. We then aim to train the entire network based on patches with both layer and nuclear annotations (model: HoVer-Net+_LS,All_).
3. Finally, we freeze the encoder and train the LS decoder only on the segmentation patches again (model: HoVer-Net+_LS,All,LS_)

During the second stage of training the LS branch was trained based on less layer annotations than in stage one, and thus will be overfit to the smaller sample used. Thus, the third stage aims to retrain the LS decoder again with all the layer annotations available.

We trained the HoVer-Net+ models over two phases. In phase one, only the decoder branches were trained for 20 epochs. In phase two, all branches (including the encoder) were trained for 30 epochs. A batch size of 8 and 4 on each GPU were used across these two phases respectively. In stage three, only the second phase of training was used as the encoder was kept frozen. For all training, the Adam optimiser was used with a learning rate that decayed initially from 10^-4^ to 10^-5^ after 10 epochs in each phase. During training, we applied the following random data augmentations: flip, rotation, Gaussian blur, median blur, and colour perturbation. We additionally, tested the effect of stain augmentation during our experiments, using the TIAToolbox (4) implementation, a method that has been shown to effectively counter scanner-induced domain-shifts, in order to create more generalisable models (5–7).

Comparative experiments were performed to test HoVer-Net+ model against other state-of-the-art models for nuclear segmentation and classification. For layer segmentation, we compared HoVer-Net+ to U-Net (8), a state-of-the-art model for semantic segmentation, following stage one of layer segmentation training. We additionally tested the performance of HoVer-Net+ for layer segmentation following further training on nuclear segmentation/classification. For nuclear segmentation/classification we further compared HoVer-Net+ to HoVer-Net (9) and U-Net (8). These models were trained based on their default parameters. For these comparisons, HoVer-Net and HoVer-Net+ were pretrained on PanNuke (10), and U-Net was pretrained on ImageNet. We further aimed to determine the generalizability of our HoVer-Net+ model to new datasets and different domain-invariance techniques.

**Supplementary Results**

**Layer and Nuclear Segmentation**

We aimed to test the generalisability of our proposed HoVer-Net+ model to new datasets in the presence of a domain shift (e.g. by scanner/site), when trained for layer segmentation alone. The data in C1 were collected on an Aperio scanner, whilst the data in C2 were predominantly collected on a Hamamatsu scanner. Thus, we expect a certain level of domain-shift between these cohorts. Our initial experiments therefore aimed to test our method, when trained/tested on combinations of the two Sheffield cohorts with annotations (C1 and C2). We also tested the effect of using different pretrained models (e.g. PanNuke (10) or ImageNet), and stain augmentation. The results for these experiments are displayed in Supplementary Table 3. Training on both cohorts generally improved performance across both datasets, whereas stain augmentation was seen to not necessarily be beneficial. Overall, trial 5 gained the best overall F1-score, when pretrained on PanNuke and trained on both C1 and C2, without any stain augmentation. We suggest that when both datasets are present within both the train and test set, the model learns enough variation over the different scanners used, and thus stain augmentation is not necessarily useful.

Following optimisation, our HoVer-Net+ model (trained for layer segmentation) was compared with U-Net (no stain augmentation, trained on C1 and C2). The results for these comparative experiments can also be seen in Supplementary Table 4. Here, multiple trials of HoVer-Net+ are displayed. First, HoVer-Net+_LS_ describes the model trained for layer segmentation alone. Unsurprisingly, this method achieved the best F1-score of 0.819, whilst U-Net (ResNet-50 encoder) achieved the worst results for layer segmentation. We additionally make further comparisons, showing how the layer segmentation performance changes with the additional training of the HoVer-Net+ model with nuclear information. HoVer-Net+_LS,All_ describes the model in its second stage of training. Following on from the first stage, where the model was trained for layer segmentation alone, the second stage describes the model that is then trained to perform simultaneous nuclear instance segmentation/classification and layer segmentation. Intuitively, there is a slight drop in the model performance, with incorporation of nuclear information, as there are less patches with both nuclear and layer information, and thus the model will be slightly overfitted to this smaller sample size. However, we combated this by introducing a third stage of training, where we froze the encoder, and the nuclear instance segmentation/classification decoder branches, and further tuned the LS decoder branch on the entire cohort of layer patches (HoVer-Net+_LS,All,LS_). This acted to improve layer segmentation only slightly when compared to HoVer-Net+_LS,All_.

The HoVer-Net+ results for nuclear instance segmentation and classification are included in Supplementary Table 5; where we have additionally compared the performance against HoVer-Net (9) and U-Net (8). U-Net has expectedly gained the worst performance, owing to its inability to differentiate touching nuclei well. This is best illustrated in Supplementary Figure 2, where touching basal layer epithelial nuclei are poorly discriminated. Within this table, HoVer-Net+_LS,All_ was initially trained on all layer segmentation data, and then further trained on all nuclei/layer data. Following the training of HoVer-Net+ on nuclei and layer information (e.g. HoVer-Net+_LS,All_), unsurprisingly there is a decrease in performance for layer segmentation (see Supplementary Table 5). The mean F1-score for HoVer-Net+ decreases from 0.819 to 0.812 owing to there being much fewer patches with both layer and nuclear annotations. We therefore opted to further finetune the HoVer-Net+ models (LS decoder only) on the entire cohort of layer patches (stage three of training). Following finetuning, the mean F1-score of HoVer-Net+ (HoVer-Net+_LS,All,LS_ in Supplementary Table 5) only increased marginally from 0.812 to 0.813. Since, this stage was done with the LS decoders alone, this should have little effect on the performance of HoVer-Net+ on nuclear segmentation/classification, only really effecting the nuclear classification performance. As can be seen from Supplementary Table 5 this didn’t affect the segmentation/detection quality of HoVer-Net+_LS,All,LS_; however, did act to improve with the classification of nuclei. This demonstrates the benefits of using HoVer-Net+ in a multi-task learning framework. When we compare both of our HoVer-Net+ models to HoVer-Net we see an improved performance across both datasets, showing the superiority of the method over both cohorts C1 and C2. Thus, this model is used for inference on all WSIs in both the internal and external datasets seen in the next step of our analysis pipeline.

The output layer and nuclear segmentations of HoVer-Net+ on a ROI from the test set are displayed in Supplementary Figure 2. The top row shows the GT layer segmentation on the left followed by HoVer-Net+ (HoVer-Net+_LS,All,LS_) and U-Net. Here, we can see that HoVer-Net+ appears to have the best quality segmentation. The blue boxes show areas where HoVer-Net+ has segmented regions that the GT segmentation has misclassified. In general, U-Net’s segmentations appear more spurious. The bottom row shows the nuclear segmentations for the GT, HoVer-Net+, HoVer-Net and U-Net (from left to right). Both HoVer-Net and HoVer-Net+ have incorrectly classified some epithelial nuclei as keratin nuclei, however, HoVer-Net appears to have misclassified many more of these cases, demonstrating the benefits of including the LS decoder branch.

We finally, show sample HoVer-Net+ nuclear segmentation/classification output on both the internal Sheffield and external cohorts in Supplementary Figure 3. Generally, the nuclear classification seems robust on the internal cohort, however, there are some errors in the external cohort where there are some misclassification of nuclear vs “other” nuclei (specifically within the lower images).

**Added Value of HoVer-Net+**

In the first stage of our experiment, we tested the performance and generalisability of our HoVer-Net+ method to new cohorts and domain invariance techniques. HoVer-Net+, when trained for layer segmentation, achieved the best results when pretrained on PanNuke over ImageNet. This is perhaps unsurprising, demonstrating the merits of pretraining on domain-specific images. We additionally tested how well HoVer-Net+ performed when trained/tested on different datasets. Overall, the mean F1-score across both C1 and C2 (used in training HoVer-Net+) was highest when trained over both datasets. Counterintuitively, we also found stain augmentation to have mixed effects on model performance, even when testing on new data. Since, we wished to generate a model that generalised as much as possible to new datasets we used the model trained on both datasets going forward, without any stain augmentation.

We performed multiple experiments testing the order of training HoVer-Net+ for each of the different tasks. We first trained the model on just layer segmentation data, as there were many more layer annotations than nuclear annotations. Next, we further trained the HoVer-Net+ model for nuclear segmentation/classification and layer segmentation (NP, HoVer, NC decoders pretrained on PanNuke, LS decoder trained in previous stage). Following this training (labelled HoVer-Net+_LS,All_), there was an expected reduction in layer segmentation performance, owing to the encoder and LS decoder being further trained on a much smaller subset of data, and therefore being more susceptible to overfitting. We therefore circumvented this issue, by freezing the encoder and the nuclear decoder branches (NP, HoVer, NC), and finetuning the LS decoder branch on the entire layer segmentation data. This then resulted in an improved performance for layer segmentation (labelled HoVer-Net+_LS,All,LS_) when compared to the HoVer-Net+_LS,All_. Since the nuclear decoder branches were not altered here, this did not affect the nuclear segmentation performance of the model; but did improve the performance for nuclear sub-classification of epithelial nuclear types (e.g. as basal, epithelial, keratin), which is dependent on the output of the LS branch. This demonstrates the merits of our multi-task learning approach, where training over multiple tasks can improve the representation of the images learned by the encoder, to benefit both tasks.

Following the optimisation of HoVer-Net+ we compared its performance to U-Net for layer segmentation. Here, we found HoVer-Net+ to get the best performance, with U-Net being worse. We additionally compared HoVer-Net+ to HoVer-Net and U-Net for nuclear segmentation/classification, where we found HoVer-Net+ to gain the best results. Overall, these results express the benefits of our multi-task learning HoVer-Net+ method, which we expect to generalise well to new data.

**Slide-level Transformation Prediction with our MLP**

In this section, we aimed to perform the feature analyses based on both the internal and external validation sets. This included first visually studying the most predictive patches, before then determining differences in the cellular composition and morphology of these patches. Following this, we use Random Forests to determine feature importances, and partial dependency plots to test the effect of certain features on the predicted outcome. Finally, we explore additional features that may prove useful in future studies, such as mitosis.

We display a randomly generated selection of the top five most predictive patches for TP cases and top five patches from TN cases on internal validation in Supplementary Figure 4. Visually, it is clear that there appear to be a larger number of immune cells both within the epithelium and the connective tissue within the TP cases.

Next, we discuss the feature analysis based on the internal cohorts (see main manuscript for external results). Patch-level nuclear counts revealed higher cellularity in true positive (TP) patches compared to true negatives (TNs) (Cohen’s *d* = 0.70, *p* < 0.001; see Supplementary Figure 5, Cellular Composition: Entire Patch), primarily driven by “other nuclei” in TPs (*d* = 2.26, *p* < 0.001). In contrast, there were slightly more epithelial cells in TNs (labelled as “All Epith” in Supplementary Figure 5; *d* = 0.70, *p* < 0.001). The higher number of “other nuclei” in these patches may be due to an increase in the number of intra-epithelial lymphocytes and peri-epithelial lymphocytes. When focussing on the nuclear counts within the epithelial region of the patch alone (Supplementary Figure 5, Cellular Composition: Epithelium), significant differences were found in the number of “other” nuclei within the epithelium (*d* = 2.29, *p* < 0.001), confirming the disparity in the number of intra-epithelial lymphocytes. Additionally, there were more epithelial nuclei within the epithelial layer in TNs (*d* = 1.31, *p* < 0.001), while more basal epithelial nuclei were observed in TPs (*d* = 0.24, *p* < 0.001). No difference was found in the number of keratin nuclei between classes (*d* = 0.03, *p =* 0.50). Lastly, Supplementary Figure 5 (Cellular Composition: Connective Tissue) illustrates a larger number of “other” nuclei within the connective tissue of TPs compared to TNs (*d* = 1.73, *p* < 0.001), confirming the increased presence of peri-epithelial lymphocytes in TPs.

When analysing the distributions of tissue types (or morphology) within patches (Supplementary Figure 5, Morphology: Entire Patch), we found that TP patches had a higher ratio of connective tissue (presumed from “other” tissue) compared to TNs (*d* = 2.22, *p* < 0.001). This is consistent with the prior nuclear analysis showing more “other” nuclei in TP patches. Additionally, TP patches often had more basal tissue (*d* = 1.02, *p* < 0.001), but less epithelial tissue (*d* = 1.33, *p* < 0.001), compared to TNs. Interestingly, TNs had significantly more surface keratin compared to TPs (*d* = 0.10, *p* = 0.02). TN patches primarily focussed on the epithelium, with little emphasis on the connective tissue, while TP patches showed a focus on the basal layer and connective tissue.

We additionally aimed to determine which of the 168 nuclear features used to train our MLP were most important for making the final prediction (on both internal and external validation). Thus, we trained a Random Forest classifier based on the 168 nuclear features in the top five correctly predicted patches by our MLP model (per correctly predicted case). We then ranked the feature importance (mean decrease in impurity, MDI), selected the top ten features and performed two-tailed t-tests (with FDR correction), to determine statistical significance. We present the top ten features found from this analysis (using MDI importance), for internal validation in Supplementary Figure 6a. Spatial features were generally estimated to be most important and made up the top ten features. We further display boxplots showing the distribution of the top five of these features in in Supplementary Figure 6b and provide results from a two-tail t-test (following FDR correction). The minimum number of “other” nuclei surrounding an epithelial nucleus within 400 microns had the highest importance, although all of the top ten features had large effect sizes (*d* > 2.00) and were shown to be significant (*p* < 0.001). These top features suggest the importance of lymphocytes (e.g. “other” nuclei) and their proximity to epithelial nuclei (e.g. intra-epithelial/peri-epithelial lymphocytes).

We further replicate these analyses on external validation. Interestingly, we note, that on external validation the top ten features are an equal mixture of both morphological and spatial features (see Supplementary Figure 7a). There is an overlap in top predictive features in internal and external validation as listed below:

1. Standard Deviation in the number of “other” nuclei surrounding “other” nuclei within 400 microns
2. Standard Deviation in the number of “other” nuclei surrounding “other” nuclei within 300 microns
3. Minimum number of “other” nuclei surrounding “epithelial” nuclei within 200 microns
4. Maximum number of “other” nuclei surrounding “other” nuclei within 300 microns

We further display boxplots showing the distribution of the top five of these features in in Supplementary Figure 7b and provide results from a two-tail t-test (following FDR correction). The standard deviation of the number of “other” nuclei surrounding an epithelial nucleus within 400 microns had the highest importance, although all of the top ten features had large effect sizes (*d* > 1.00) and were shown to be significant (*p* < 0.001). These top features again suggest the importance of lymphocytes (e.g. “other” nuclei) and their proximity to epithelial nuclei (e.g. intra-epithelial/peri-epithelial lymphocytes). However, on external validation, we additionally see the importance of the (variation in) size of “other” nuclei (given by convex area, diameter, perimeter, and bounding box area).

To further validate the findings of the hotspot-coldspot feature importance, we additionally explore the partial dependency probability plots for our MLP model when tested on external cases. In this analysis, we systematically varied each of the 168 input features, on a feature-by-feature basis, for all cases in the external test set. We varied the value of each feature from their minimum value to the their maximum value over 100 increments, and plotted this against the predicted probability of the model output over all cases. We give nine of the features which visually appeared to have the largest gradients (i.e steepest change) in Supplementary Figure 8. Within the top row, these plots shows clear relationships between larger maximum major axis lengths and convex areas in “other” nuclei, as well as the standard deviation in the major axis length in “other” nuclei, and malignant transformation. We see in the middle row the positive relationship between the maximum area (bounding box and convex area), and the major axis length of epithelial nuclei, and malignant transformation. Finally, in the bottom row, we see a positive correlation between higher amounts of “other” nuclei surrounding epithelial nuclei.

Overall, we see strong positive relationships between morphological features, concerning both epithelial and “other” nuclei, and malignant transformation. These results demonstrate the importance of the predictive potential of changes in the shape and size of nuclei, as specified as a cytological feature in the OED WHO grading system (e.g. anisonucleosis, nuclear pleomorphism). However, these plots also further suggest the potential importance of both PELs and EILs, where there is a clear relationship between the mean/maximum number of “other” nuclei surrounding an epithelial nucleus, within varying radii.

Finally, we aimed to explore additional features that may add predictive power to our models. We did not incorporate any mitotic features into our models, however, we additionally aimed to determine whether our top predicted patches could further inform us of their importance. We therefore, ran the MIDOG 2022 winning code (available online: [adamshephard/TIA-mitosis (github.com)](https://github.com/adamshephard/TIA-mitosis)) on the top predicted patches from our analyses (internal validation alone, for feature discovery). Since mitotic figures are generally sparse in dysplasia, we generated a mitotic count per slide over all of the five top predicted patches by the model (in TP vs TN cases, respectively). Only slides that included one or more mitotic figures were included in this analysis. A two-tailed t-test was then used to determine statistical significance.

The MIDOG 22 winning model appeared to work well on our dataset (by visual inspection). However, we found no difference between the number of mitotic figures discovered in TPs vs TNs (*d* = 0.06, *p* = 0.82). This was perhaps an unsurprising finding, as mitotic figures were not included as a feature in our MLP models.

**External Validation Exclusion of Poor Segmentations with our MLP**

We additionally aimed to test the performance of our MLP model, on external validation, with the exclusion of cases that HoVer-Net+’s nuclear/region segmentation results were deemed insufficient quality (*n* = 13). Here, segmentations were deemed as insufficient quality if either a) a substantial portion of connective tissue was incorrectly segmented as epithelium (i.e. false positives), or b) a substantial portion of the epithelium tissue was not segmented as epithelium (i.e. false negatives), by visual assessment. We performed this analysis to determine how epithelium segmentation quality effected the overall pipeline, and to inform us whether an improved quality HoVer-Net+ might lead to better transformation prediction. The results of this analyses are shown in Supplementary Table 6. We would like to add here that all excluded cases were from the Birmingham dataset, in this analysis. We see a clear increase in performance compared to without the quality control (QC). This shows the importance of having a robust segmentation pipeline for this analysis. With training on more diverse data, we expect HoVer-Net+ to generalise better to unseen datasets.

**Comparison to ResNet34-IDaRS Method for Transformation Prediction**

We additionally compared our MLP method for transformation prediction to another state-of-the-art method. For this comparison, we train a ResNet-34 model using iterative draw-and-rank sampling (IDaRS), on image patches from the epithelium alone, to predict transformation, thus replicating the original IDaRS pipeline for our transformation prediction problem. We will label this as ResNet34-IDaRS from here on. We suggest that one of the main advantages of our MLP trained with morphological/spatial features, is that these features are in theory “domain-agnostic”. Thus, providing the deep learning models used to generate the nuclear segmentations generalise, then these features should generalise well to new, unseen datasets. In contrast, we suggest that deep models such as ResNet34-IDaRS may not generalise well to unseen data. Thus, we have additionally employed domain adaption techniques to make the ResNet34-IDaRS model robust to new data, in order to make a fair-comparison to our MLP. Thus, we additionally include the ResNet34-IDaRS model, trained using a) Macenko stain augmentation, b) domain adversarial training, and c) a combination of these two techniques. Domain adversarial training was introduced by Ganin *et al.* (2016) (12), and aims to force the encoder part of the CNN to learn domain-agnostic features. Within this work, an additional classifier head is added to the ResNet34-IDaRS model that aims to predict the domain from which the provided features are from (in this case, the scanner used). The addition of a gradient reversal layer aims to maximise the error in this classification task, thus incentivising the classification model to learn domain-agnostic features.

In all models presented in this work, the patches used for feature generation are based on HoVer-Net+’s segmentation of the epithelium. All models were additionally trained with a symmetric cross-entropy loss function, and the Adam optimiser. We chose the parameters *k* = 5 top predictive patches and *r =* 45 random patches in the iterative draw-and-rank method, with a batch size of 256. All models were trained until convergence with a minimum of 30 and a maximum of 100 epochs.

In these experiments (see Supplementary Table 7 and Supplementary Table 8), we have compared the performance of our MLP to the ResNet34-IDaRS model. Overall, the results tell an interesting story, with relation to domain adaption. The raw ResNet34-IDaRS model that obtained high results on internal cross-validation (AUROC = 0.78), has not generalised well to the new unseen data (AUROC = 0.51). The various domain adaption techniques, such as domain adversarial training and stain augmentation, did not help to improve model performance on internal validation; but, helped improve the generalisability of the ResNet34-IDaRS model on external validation. However, ultimately, the model that was trained with domain-agnostic features (e.g. our MLP) achieved the highest results on external validation (AUROC = 0.75).

ResNet34-IDaRS generated predictions based on deep features learned through training (here, ResNet-34). This model differs from the MLP pipeline used primarily in this study, in that the features that are used in the MLP are deliberately generated, with the purpose of being interpretable and important. However, the ResNet34-IDaRS framework does generate good predictions for internal validation. Thus, we also explored the hot/coldspots found by the ResNet34-IDaRS model (on internal validation, ResNet34-IDaRS-DA-SA) to determine what were the important features used for prediction. Interestingly, we found a significantly higher number of mitotic figures in the TP patches than the TN patches (*d* = 0.63, *p* = 0.01; see Supplementary Figure 9). This suggests that the ResNet34-IDaRS model has independently learned to account for mitosis as a deep feature.

**Supplementary Discussion**

**The Difficulty of the Task**

In this work, we aimed to generate a model that would better predict transformation (our *OMTscore*), using interpretable features. We also wished to validate this model on external cohorts. However, some comments must be included on the difficulty of this task. OED data can be relatively sparse, and we therefore found it difficult to collate large external cohorts. Even with the inclusion of the Birmingham and Belfast data, we found that the survival information of these cohorts varied drastically, adding to the difficulty of the task. We have therefore included the independent survival curves of our cohorts in Supplementary Figure 10. Here, we can see how the survival curves for these cohorts vary drastically, with low concordance and a significant difference via a log-rank test. This confounding variable is the clinical reality, and thus adds to the strength of our model’s performance.

**Domain Generalisation and Future Work**

In this work, we aimed to generate a model that would better predict transformation, using interpretable features. Ultimately, these morphological/spatial features were based on shape/size differences (e.g. the differences in the mean radius of nuclei in a patch) in different nuclear types (e.g. epithelial vs “other” nuclei). Overall, we found our MLP model to gain the best results for predicting transformation on external validation. Interestingly, despite the success of ResNet34-IDaRS with deep features in the past, we have found that by instead using morphological/spatial features one can achieve better performance. We also argue that the incorporation of such features in favour of deep features, makes our models more interpretable. ResNet34-IDaRS, which initially gained the highest AUROC values on internal validation, failed to generalise to the new, unseen data without any form of domain alignment/augmentation techniques. As was expected, the addition of stain augmentation and domain adversarial training, improved the performance of ResNet34-IDaRS on new data (from AUROC = 0.51 to AUROC = 0.76). However, ultimately our MLP model, trained on interpretable morphological/spatial features gained the highest performance in terms of both AUROC and F1-score (AUROC = 0.75; F1 = 0.69). The F1-scores of the ResNet34-IDaRS-based models were generally low, suggesting a lack of model convergence. Our MLP model features are essentially domain-agnostic (when segmented accurately), and we suggest that this is the reason for their superior performance.

Despite this, we still explored the hotspot/coldspot regions produced by ResNet34-IDaRS, and found that mitoses potentially contributed to the model performance. Future work should look at incorporating tile-level (or even slide-level) mitotic scoring into the MLP model, to further improve the performance.

**Supplementary References**

1. Alemi Koohbanani N, Jahanifar M, Zamani Tajadin N, Rajpoot N. NuClick: A deep learning framework for interactive segmentation of microscopic images. Med Image Anal. 2020;65.

2. Jahanifar M, Koohbanani NA, Rajpoot N. NuClick: From Clicks in the Nuclei to Nuclear Boundaries. arXiv [Internet]. 2019;(Dl). Available from: http://arxiv.org/abs/1909.03253

3. Graham S, Vu QD, Jahanifar M, Raza SEA, Minhas F, Snead D, et al. One model is all you need: Multi-task learning enables simultaneous histology image segmentation and classification. Med Image Anal. 2023;83(October 2022).

4. Pocock J, Graham S, Vu QD, Jahanifar M, Deshpande S, Hadjigeorghiou G, et al. TIAToolbox as an end-to-end library for advanced tissue image analytics. Commun Med [Internet]. 2022;2(1):120. Available from: https://www.nature.com/articles/s43856-022-00186-5

5. Aubreville M, Stathonikos N, Bertram CA, Klopfleisch R, ter Hoeve N, Ciompi F, et al. Mitosis domain generalization in histopathology images — The MIDOG challenge [Internet]. Vol. 84, Medical Image Analysis. Elsevier; 2023 [cited 2023 Jan 6]. p. 102699. Available from: http://arxiv.org/abs/2204.03742

6. Jahanifar M, Shephard A, Tajeddin NZ, Bashir RMS, Bilal M, Khurram SA, et al. Stain-Robust Mitotic Figure Detection for the Mitosis Domain Generalization Challenge. arXiv [Internet]. 2021;3–5. Available from: http://arxiv.org/abs/2109.00853

7. Jahanifar M, Shephard A, Zamanitajeddin N, Raza SEA, Rajpoot N. Stain-Robust Mitotic Figure Detection for MIDOG 2022 Challenge. 2022;1–3. Available from: http://arxiv.org/abs/2208.12587

8. Ronneberger O, Fischer P, Brox T. U-net: Convolutional networks for biomedical image segmentation. Lect Notes Comput Sci (including Subser Lect Notes Artif Intell Lect Notes Bioinformatics). 2015;9351:234–41.

9. Graham S, Vu QD, Raza SEA, Azam A, Tsang YW, Kwak JT, et al. Hover-Net: Simultaneous segmentation and classification of nuclei in multi-tissue histology images. Med Image Anal [Internet]. 2019;58:101563. Available from: https://doi.org/10.1016/j.media.2019.101563

10. Gamper J, Koohbanani NA, Benes K, Graham S, Jahanifar M, Khurram SA, et al. PanNuke Dataset Extension, Insights and Baselines. 2020;1–12. Available from: http://arxiv.org/abs/2003.10778

11. Bilal M, Raza SEA, Azam A, Graham S, Ilyas M, Cree IA, et al. Development and validation of a weakly supervised deep learning framework to predict the status of molecular pathways and key mutations in colorectal cancer from routine histology images: a retrospective study. Lancet Digit Heal [Internet]. 2021;3(12):e763–72. Available from: http://dx.doi.org/10.1016/S2589-7500(21)00180-1

12. Ganin Y, Ustinova E, Ajakan H, Germain P, Larochelle H, Laviolette F, et al. Domain-adversarial training of neural networks. In: Advances in Computer Vision and Pattern Recognition. 2017. p. 189–209.

**Supplementary Tables**

Supplementary Table 1
*Demographic, characteristic and outcome data for the OED cases from Sheffield, Birmingham and Belfast cohorts.*

|  | Sheffield | Belfast | Birmingham |
| --- | --- | --- | --- |
| OED Cases*, n* | 193 | 42 | 47 |
| OED Slides*, n* | 270 | 42 | 47 |
| Median Age^a^ (IQR) | 64 (55 – 74) | 62 (52 – 71) | 61 (51 – 70) |
| Sex^b^, *n* (%) |  |  |  |
| Female | 124 (46) | 21 (50) | 24 (51) |
| Male | 145 (54) | 21 (50) | 23 (49) |
| Site, (%) |  |  |  |
| Buccal Mucosa | 35 (13) | 0 (0) | 6 (13) |
| Tongue | 118 (44) | 29 (69) | 30 (64) |
| Floor of Mouth | 57 (21) | 9 (21) | 3 (6) |
| Other | 60 (22) | 4 (10) | 8 (17) |
| WHO grade, *n* (%) |  |  |  |
| Mild | 91 (34) | 6 (14) | 24 (51) |
| Moderate | 104 (39) | 25 (60) | 18 (38) |
| Severe | 75 (28) | 11 (26) | 5 (11) |
| Binary grade, *n* (%) |  |  |  |
| Low-risk | 169 (63) | 7 (17) | 28 (60) |
| High-risk | 101 (37) | 35 (83) | 19 (40) |
| Transformation, *n* (%) | 57 (21) | 30 (71) | 10 (21) |
| Median Follow-up Time, *months* (IQR) | 95 (57 – 108) | 42 (23 – 74) | 47 (32 – 66) |
| Scanner, *n* (%) |  |  |  |
| Aperio CS2 | 173 (64) | 0 (0) | 0 (0) |
| Hamamatsu NanoZoomer 360 | 97 (36) | 0 (0) | 0 (0) |
| Aperio AT2 | 0 (0) | 42 (100) | 0 (0) |
| Pannoramic 250 | 0 (0) | 0 (0) | 47 (100) |

*Note.* *All provided statistics are at the slide-level (not patient-level) since one patient may have had multiple OED lesions and thus have different clinical information associated to the slide, i.e. Age at Diagnosis, Site, Grade.*

*^a^ Median age at diagnosis of OED.*

*^b^ One Sheffield patient had no information regarding sex available.*

Supplementary Table 2
*Sheffield cohorts used for training HoVer-Net+.*

|  | Layers | | Nuclei | |
| --- | --- | --- | --- | --- |
|  | Cohort 1 | Cohort 2 | Cohort 1 | Cohort 2 |
| Cases, *n* |  |  |  |  |
| Aperio CS2 | 38 | 0 | 20 | 0 |
| Hamamatsu NanoZoomer 360 | 0 | 21 | 0 | 10 |
| Controls, *n* |  |  |  |  |
| Aperio CS2 | 5 | 4 | 0 | 0 |
| Hamamatsu NanoZoomer 360 | 0 | 0 | 0 | 0 |

Supplementary Table 3
*Experiments for layer segmentation on both dataset 1 and 2, based on F1-scores.*

| Trial | Train Set | Pretrain. | Stain.  Aug. | Test C1 | | | | | | Test C2 | | | | | | Overall Mean |
| --- | --- | --- | --- | --- | --- | --- | --- | --- | --- | --- | --- | --- | --- | --- | --- | --- |
|  |  |  |  | Bkgd. | Other | Basal | Epith. | Keratin | Mean | Bkgd. | Other | Basal | Epith. | Keratin | Mean |  |
| 1 | C2 | PanNuke | N | 0.872 | 0.765 | 0.563 | 0.802 | 0.644 | 0.729 | 0.910 | **0.853** | **0.771** | 0.888 | 0.791 | 0.843 | 0.786 |
| 2 | C2 | PanNuke | Y | 0.822 | 0.713 | 0.557 | 0.739 | 0.547 | 0.675 | 0.909 | 0.828 | 0.714 | 0.861 | 0.774 | 0.817 | 0.746 |
| 3 | C1 | PanNuke | N | 0.842 | 0.807 | 0.717 | 0.819 | 0.660 | 0.769 | 0.844 | 0.813 | 0.720 | 0.851 | 0.780 | 0.802 | 0.785 |
| 4 | C1 | PanNuke | Y | 0.849 | 0.819 | 0.700 | 0.838 | 0.682 | 0.778 | 0.876 | 0.835 | 0.727 | 0.865 | 0.772 | 0.815 | 0.796 |
| **5** | **C1,C2** | **PanNuke** | **N** | 0.874 | **0.849** | **0.721** | **0.858** | **0.717** | **0.804** | 0.904 | 0.849 | 0.753 | 0.880 | 0.789 | 0.835 | **0.819** |
| 6 | C1,C2 | PanNuke | Y | 0.850 | 0.821 | 0.706 | 0.835 | 0.674 | 0.777 | 0.901 | 0.851 | 0.739 | 0.884 | **0.801** | 0.835 | 0.806 |
| 7 | C2 | ImageNet | N | 0.869 | 0.774 | 0.594 | 0.797 | 0.627 | 0.732 | **0.911** | 0.851 | 0.760 | **0.891** | 0.798 | **0.842** | 0.787 |
| 8 | C2 | ImageNet | Y | 0.843 | 0.742 | 0.544 | 0.781 | 0.603 | 0.703 | 0.910 | 0.842 | 0.695 | 0.868 | 0.776 | 0.818 | 0.760 |
| 9 | C1 | ImageNet | N | **0.878** | **0.849** | 0.715 | 0.852 | 0.698 | 0.798 | 0.864 | 0.473 | 0.533 | 0.446 | 0.251 | 0.513 | 0.656 |
| 10 | C1 | ImageNet | Y | 0.847 | 0.807 | 0.711 | 0.826 | 0.664 | 0.771 | 0.822 | 0.806 | 0.722 | 0.846 | 0.783 | 0.796 | 0.783 |
| 11 | C1,C2 | ImageNet | N | 0.864 | 0.808 | 0.701 | 0.815 | 0.654 | 0.768 | 0.905 | 0.777 | 0.714 | 0.809 | 0.578 | 0.756 | 0.762 |
| 12 | C1,C2 | ImageNet | Y | 0.817 | 0.755 | 0.672 | 0.780 | 0.577 | 0.720 | 0.888 | 0.834 | 0.713 | 0.870 | 0.784 | 0.818 | 0.769 |

Supplementary Table 4
*Comparative experiments for layer segmentation on both dataset 1 and 2, based on F1-scores.*

| Model | Pretrain. | Test C1 | | | | | | Test C2 | | | | | | Overall Mean |
| --- | --- | --- | --- | --- | --- | --- | --- | --- | --- | --- | --- | --- | --- | --- |
|  |  | Bkgd. | Other | Basal | Epith. | Keratin | Mean | Bkgd. | Other | Basal | Epith. | Keratin | Mean |  |
| U-Net | ImageNet | **0.880** | **0.849** | **0.739** | 0.853 | 0.708 | **0.806** | **0.905** | 0.777 | 0.714 | 0.809 | 0.578 | 0.756 | 0.781 |
| HoVer-Net+_LS_ | PanNuke | 0.874 | **0.849** | 0.721 | **0.858** | **0.717** | 0.804 | 0.904 | **0.849** | 0.753 | 0.880 | 0.789 | 0.835 | **0.819** |
| HoVer-Net+_LS,All_ | PanNuke | 0.862 | 0.837 | 0.722 | 0.846 | 0.707 | 0.795 | 0.902 | 0.834 | 0.759 | 0.872 | 0.780 | 0.829 | 0.812 |
| **HoVer-Net+_LS,All,LS_** | PanNuke | 0.853 | 0.827 | 0.716 | 0.851 | 0.700 | 0.789 | 0.898 | **0.849** | **0.764** | **0.885** | **0.792** | **0.837** | 0.813 |
| *Note. HoVer-Net+_LS_ was trained on layer data only. HoVer-Net+_LS,All_ was trained on layer data only, then patches with both nuclei and layer data only. HoVer-Net+_LS,All,LS_ was trained on layer data only, then patches with both nuclei and layer data only, finally, the LS decoder alone was finetuned on all layer data.* | | | | | | | | | | | | | | |
|  | | | | | | | | | | | | | | |

Supplementary Table 5
*Comparative experiments for nuclear instance segmentation and classification.*

| Model | Test C1 | | | | | | | | | Test C2 | | | | | | | | |  |
| --- | --- | --- | --- | --- | --- | --- | --- | --- | --- | --- | --- | --- | --- | --- | --- | --- | --- | --- | --- |
|  | Dice | AJI | DQ | SQ | PQ | F^d^ | F_c_^o^ | F_c_^b^ | F_c_^e^ | Dice | AJI | DQ | SQ | PQ | F^d^ | F_c_^o^ | F_c_^b^ | F_c_^e^ | |
| U-Net | 0.605 | 0.378 | 0.473 | 0.606 | 0.311 | 0.627 | 0.585 | 0.245 | 0.529 | 0.582 | 0.354 | 0.444 | 0.581 | 0.302 | 0.652 | 0.541 | 0.275 | 0.522 | |
| HoVer-Net | 0.713 | 0.589 | 0.721 | **0.730** | **0.536** | **0.813** | 0.680 | 0.542 | 0.590 | 0.660 | 0.591 | 0.708 | **0.641** | 0.473 | 0.820 | 0.621 | 0.494 | 0.619 | |
| HoVer-Net+_LS,All_ | **0.716** | **0.598** | **0.724** | **0.730** | 0.533 | 0.810 | **0.719** | **0.599** | 0.633 | **0.665** | **0.635** | **0.753** | **0.641** | **0.478** | **0.833** | 0.715 | 0.590 | 0.646 | |
| **HoVer-Net+_LS,All, LS_** | **0.716** | **0.598** | **0.724** | **0.730** | 0.533 | 0.810 | 0.717 | 0.593 | **0.646** | **0.665** | **0.635** | **0.753** | **0.641** | **0.478** | **0.833** | **0.727** | **0.621** | **0.668** | |
| *Note. HoVer-Net+_LS,All_ was trained on layer data only, then patches with both nuclei and layer data only. HoVer-Net+_LS,All,LS_ was trained on layer data only, then patches with both nuclei and layer data only, finally, the LS decoder alone was finetuned on all layer data.* | | | | | | | | | | | | | | | | | | | |

Supplementary Table 6
*Slide-level mean (standard deviation) results for transformation prediction when trained on the Sheffield cohorts and tested on the external Birmingham-Belfast data, with QC of HoVer-Net+ output.*

|  | Birmingham (*n* = 34) | | | | Belfast (*n*  = 42) | | | | Combined (*n* = 76) | | | |
| --- | --- | --- | --- | --- | --- | --- | --- | --- | --- | --- | --- | --- |
| Model | F1-score | Recall | Fall-out | AUROC | F1-score | Recall | Fall-out | AUROC | F1-score | Recall | Fall-out | AUROC |
| **OMTscore** | 0.47 (0.03) | **0.90 (0.16)** | 0.52 (0.19) | **0.80 (0.01)** | **0.84 (0.01)** | **0.93 (0.03)** | 0.75 (0.08) | **0.71 (0.05)** | 0.73 (0.02) | **0.93 (0.06)** | 0.59 (0.16) | **0.78 (0.01)** |
| Binary Grade | **0.60** | 0.86 | **0.26** | **0.80** | 0.80 | 0.87 | 0.75 | 0.56 | **0.75** | 0.86 | 0.41 | 0.73 |
| WHO Grade G1 | 0.57 | 0.86 | 0.30 | 0.78 | 0.79 | 0.87 | 0.83 | 0.52 | 0.74 | 0.86 | 0.46 | 0.70 |
| WHO Grade G2 | 0.44 | 0.29 | 0.00 | 0.64 | 0.39 | 0.27 | **0.25** | 0.51 | 0.40 | 0.27 | **0.08** | 0.60 |
| *Note. WHO Grade G1 is mild vs moderate/severe cases, whilst WHO Grade G2 is mild/moderate vs severe cases.* | | | | | | | | | | | | |

Supplementary Table 7
*Slide-level mean (standard deviation) results for transformation prediction, on internal validation.*

|  | Sheffield (*n*  = 270) | | | |
| --- | --- | --- | --- | --- |
| Model | F1-score | Recall | Fall-out | AUROC |
| ResNet34-IDaRS | 0.59 (0.11) | **0.85 (0.17)** | 0.29 (0.16) | 0.78 (0.11) |
| ResNet34-IDaRS-SA | **0.59 (0.07)** | 0.84 (0.14) | 0.26 (0.12) | **0.80 (0.07)** |
| ResNet34-IDaRS-DA | 0.55 (0.10) | 0.75 (0.18) | 0.30 (0.14) | 0.73 (0.11) |
| ResNet34-IDaRS-SA-DA | 0.53 (0.08) | 0.77 (0.16) | 0.29 (0.09) | 0.72 (0.11) |
| **OMTscore** | 0.57 (0.08) | 0.84 (0.07) | 0.30 (0.12) | 0.77 (0.08) |
| Binary Grade | 0.51 (0.08) | 0.70 (0.09) | 0.28 (0.07) | 0.71 (0.06) |
| WHO Grade G1 | 0.46 (0.08) | 0..94 (0.07) | 0.59 (0.07) | 0.68 (0.05) |
| WHO Grade G2 | 0.34 (0.16) | 0.41 (0.19) | **0.24 (0.08)** | 0.58 (0.11) |
| *Note. WHO Grade G1 is mild vs moderate/severe cases, whilst WHO Grade G2 is mild/moderate vs severe cases.* | | | | |

Supplementary Table 8
*Slide-level mean (standard deviation) results for transformation prediction, on external validation.*

|  | Birmingham (*n*  = 47) | | | | Belfast (*n*  = 42) | | | | Combined (*n*  = 89) | | | |
| --- | --- | --- | --- | --- | --- | --- | --- | --- | --- | --- | --- | --- |
| Model | F1-score | Recall | Fall-out | AUROC | F1-score | Recall | Fall-out | AUROC | F1-score | Recall | Fall-out | AUROC |
| ResNet34-IDaRS | 0.41 (0.03) | 0.60 (0.10) | 0.35 (0.05) | 0.66 (0.04) | 0.37 (0.32) | 0.30 (0.26) | 0.28 (0.25) | 0.58 (0.01) | 0.40 (0.19) | 0.38 (0.22) | 0.33 (0.10) | 0.51 (0.09) |
| ResNet34-IDaRS-SA | 0.21 (0.19) | 0.16 (0.15) | 0.06 (0.06) | 0.66 (0.04) | 0.56 (0.24) | 0.52 (0.31) | 0.56 (0.41) | 0.52 (0.07) | 0.48 (0.23) | 0.43 (0.27) | 0.18 (0.14) | 0.74 (0.02) |
| ResNet34-IDaRS-DA | 0.17 (0.19) | 0.13 (0.15) | 0.10 (0.02) | 0.68 (0.05) | 0.59 (0.16) | 0.56 (0.25) | 0.64 (0.41) | 0.49 (0.04) | 0.50 (0.17) | 0.45 (0.22) | 0.23 (0.09) | 0.72 (0.06) |
| ResNet34-IDaRS-SA-DA | 0.00 (0.00) | 0.00 (0.00) | **0.01 (0.02)** | 0.70 (0.07) | 0.09 (0.11) | 0.06 (0.07) | **0.08 (0.14)** | 0.51 (0.05) | 0.07 (0.09) | 0.04 (0.05) | **0.03 (0.03)** | **0.76 (0.01)** |
| **OMTscore** | 0.44 (0.01) | 0.87 (0.06) | 0.57 (0.07) | 0.73 (0.01) | **0.84 (0.02)** | **0.93 (0.03)** | 0.69 (0.05) | **0.71 (0.03)** | 0.69 (0.01) | **0.92 (0.04)** | 0.60 (0.06) | 0.75 (0.01) |
| Binary Grade | 0.55 | 0.80 | 0.30 | 0.75 | 0.80 | 0.87 | 0.75 | 0.56 | **0.72** | 0.85 | 0.41 | 0.72 |
| WHO Grade G1 | **0.55** | **0.90** | 0.38 | **0.76** | 0.79 | 0.87 | 0.83 | 0.52 | 0.71 | 0.88 | 0.49 | 0.69 |
| WHO Grade G2 | 0.40 | 0.30 | 0.05 | 0.63 | 0.39 | 0.27 | 0.25 | 0.51 | 0.39 | 0.28 | 0.10 | 0.69 |
| *Note. WHO Grade G1 is mild vs moderate/severe cases, whilst WHO Grade G2 is mild/moderate vs severe cases.* | | | | | | | | | | | | |

**Supplementary Figures**


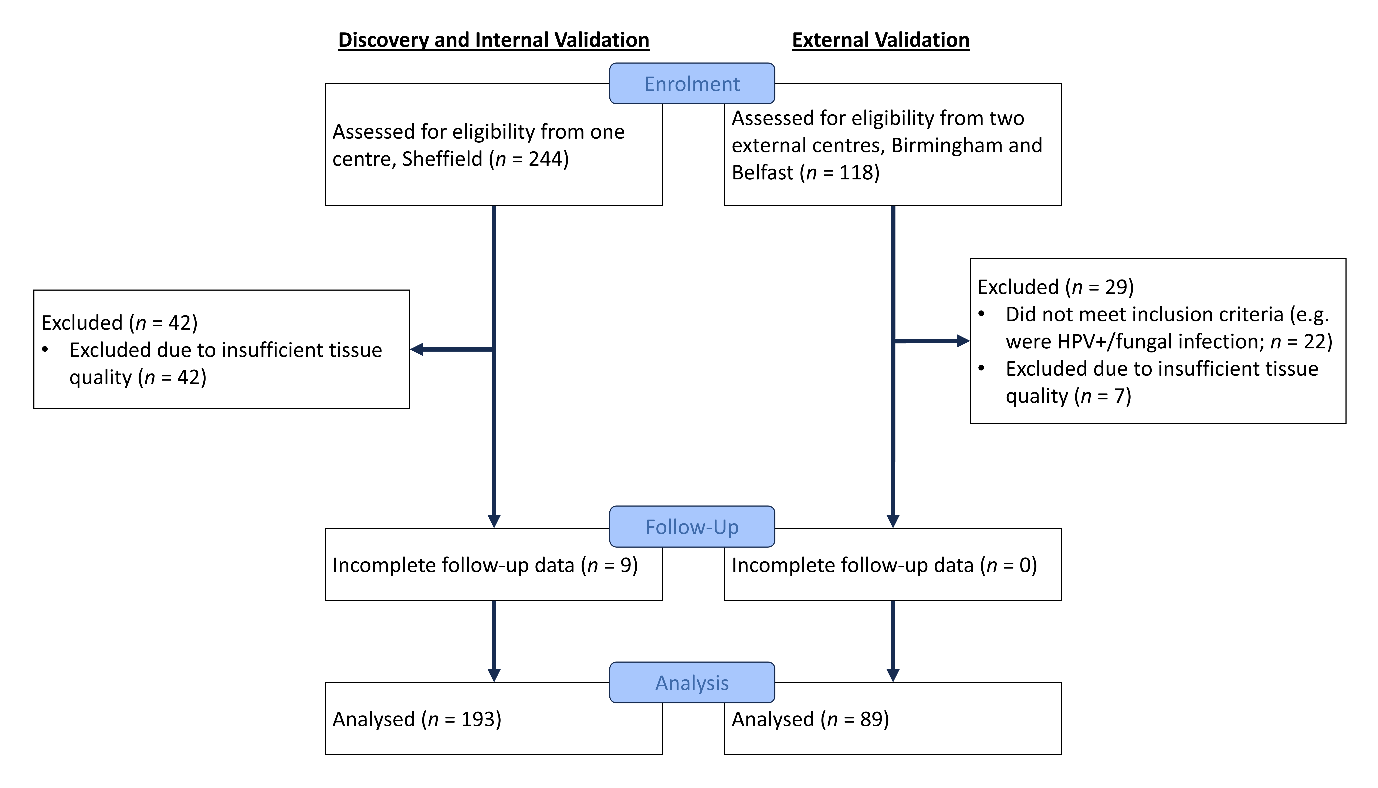


Supplementary Figure 1 CONSORT diagram for the internal and external cases used in this study. We provide a CONSORT diagram showing the cases used for discovery and internal validation on the left, and for external validation on the right. We first show all the cases enrolled to the study. We then list the cases that were further excluded owing to meeting the exclusion criteria. Cases that did not have sufficient follow-up data were not included in the final analysis.


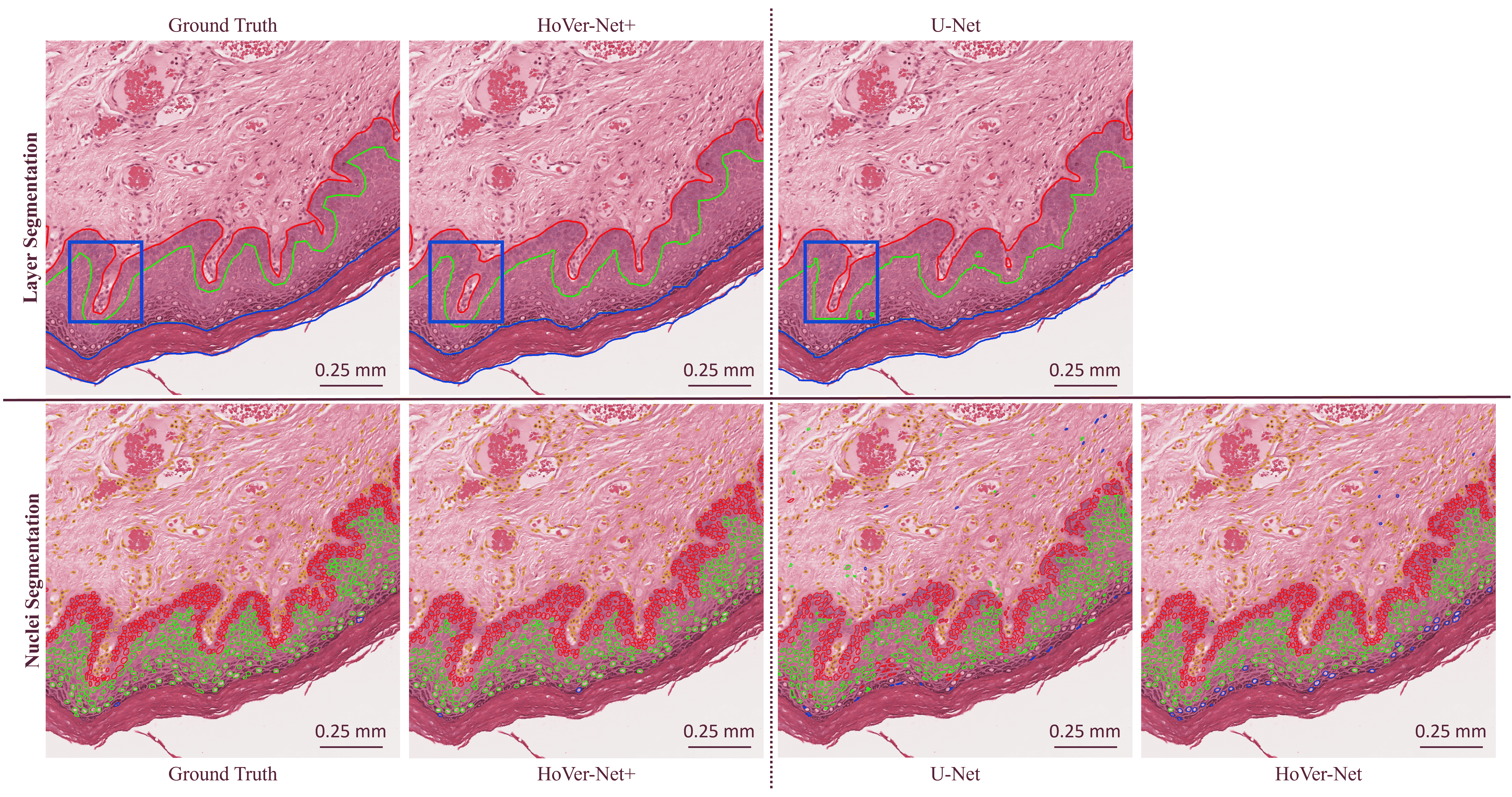


Supplementary Figure 2 Visualisation of nuclear and segmentation performance of HoVer-Net+ compared to other models. Top row: A comparison of the GT layer segmentations vs. HoVer-Net+_LS,All,LS_ and U-Net. In the images, the red line represents the basal layer, the green epithelium, and the blue keratin. For clarity, other tissue region segmentations have been excluded from these results. Bottom row: A comparison of the GT nuclear segmentations vs. HoVer-Net+_LS,All,LS_, HoVer-Net and U-Net. In the images, the red line represents the basal nuclei, green epithelial nuclei, blue keratin nuclei, orange other nuclei and finally, black represents unlabelled nuclei. Note, the dividing line between the annotations on the left and right is to display that the HoVer-Net+ segmentations for nuclei and layers are from the same model. On the right, specifically with U-Net, these are from two separately trained models.


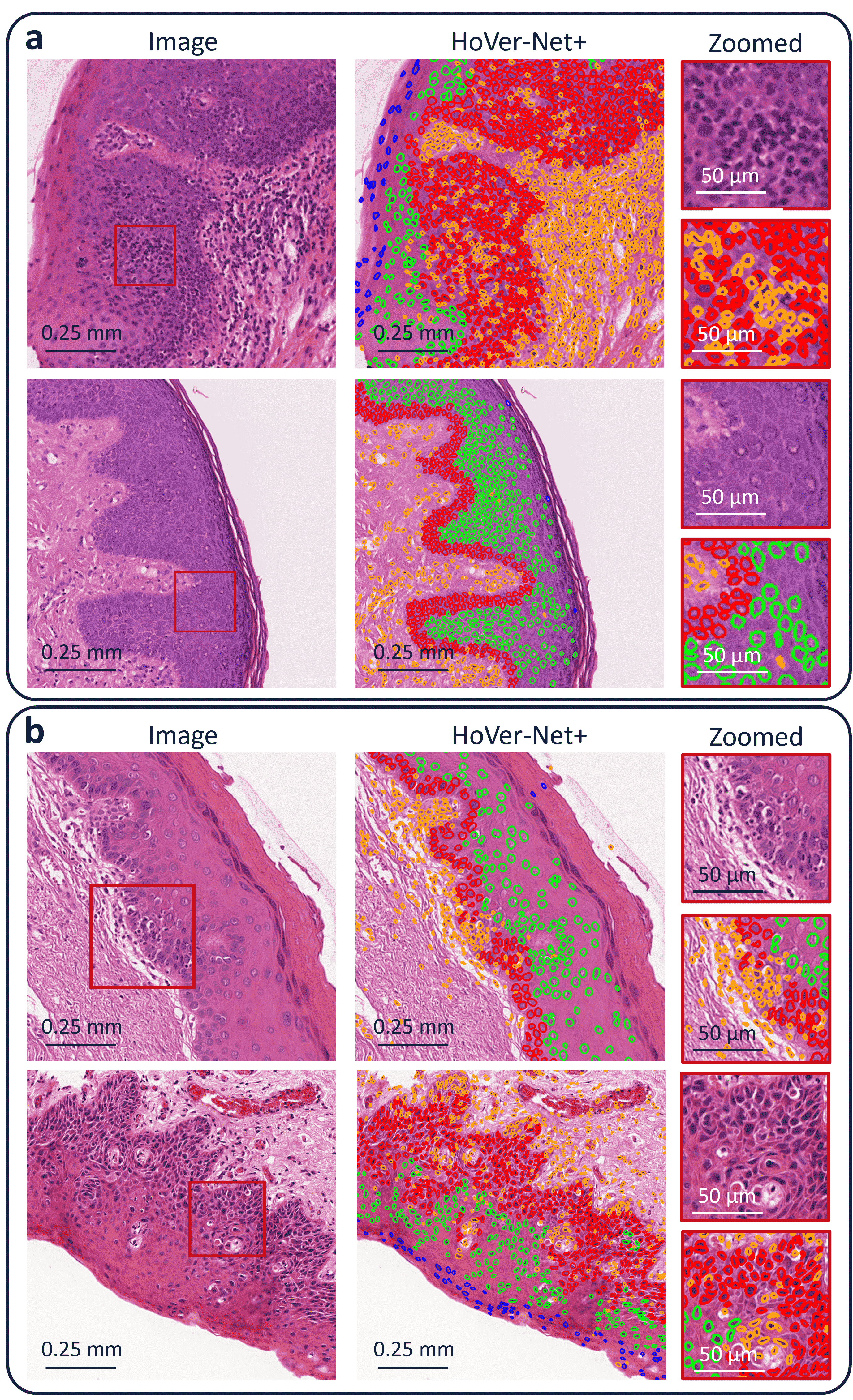


Supplementary Figure 3 Visualisation of the nuclear classification performance of HoVer-Net+. Nuclear segmentation and classification visualisations for the HoVer-Net+ output on the a internal and b external datasets. Left shows the raw image, middle shows the HoVer-Net+ output and right shows zoom-ins.


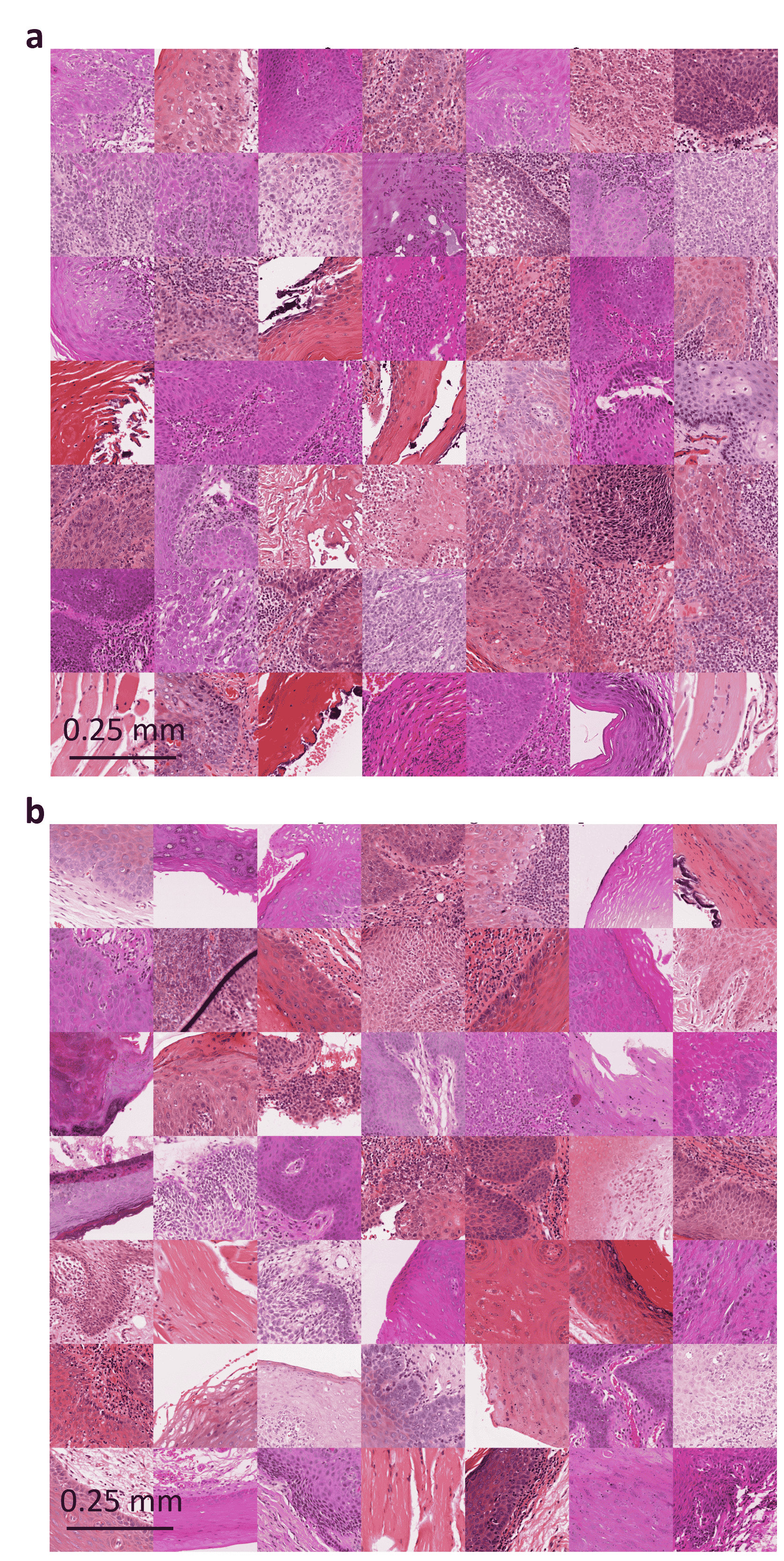


Supplementary Figure 4 Montage of top predicted patches from the MLP for predicting malignant transformation. Random patch montage of top predicted patches in a true positives and b true negatives from our MLP.


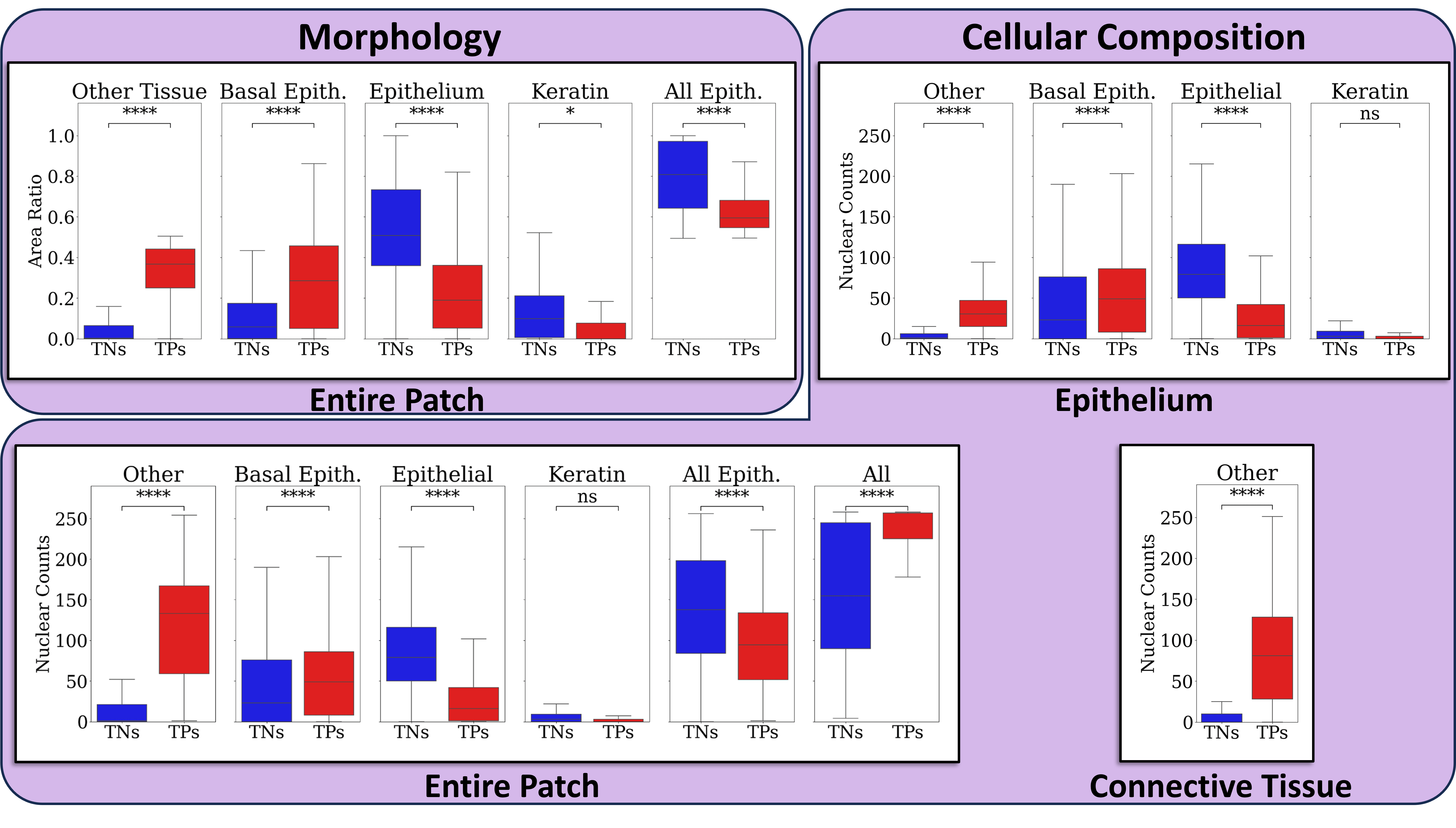


Supplementary Figure 5 Morphology and cellular composition feature analyses for internal validation. This figure shows the distribution of nuclear counts (cellular composition) within the entire patch, the epithelium alone, and the connective tissue alone, of the top five predicted patches from true positive (TP) cases, and the top five patches from true negatives (TNs), on internal validation. We additionally give boxplots showing the distribution of areas ratios (morphology) within the top five predicted patches from TPs, and the top five patches from TNs.


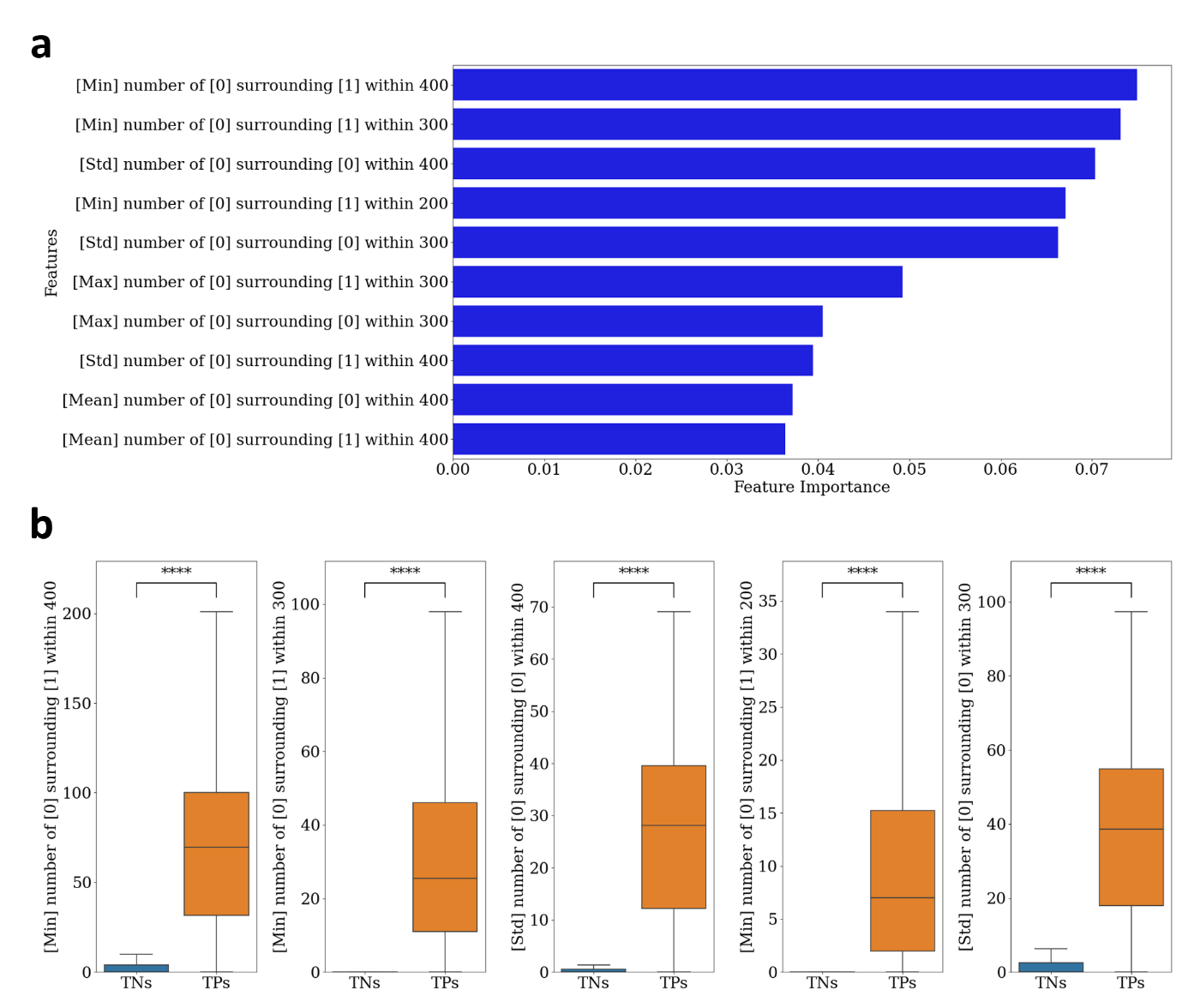


Supplementary Figure 6 Random Forest feature importance for internal validation. a The top ten most important morphological/spatial features found via a Random Forest, and b boxplots showing the distribution of the top five of these features, on internal validation. [0] are “other” nuclei, whilst [1] are epithelial nuclei. Distance is measure in microns.


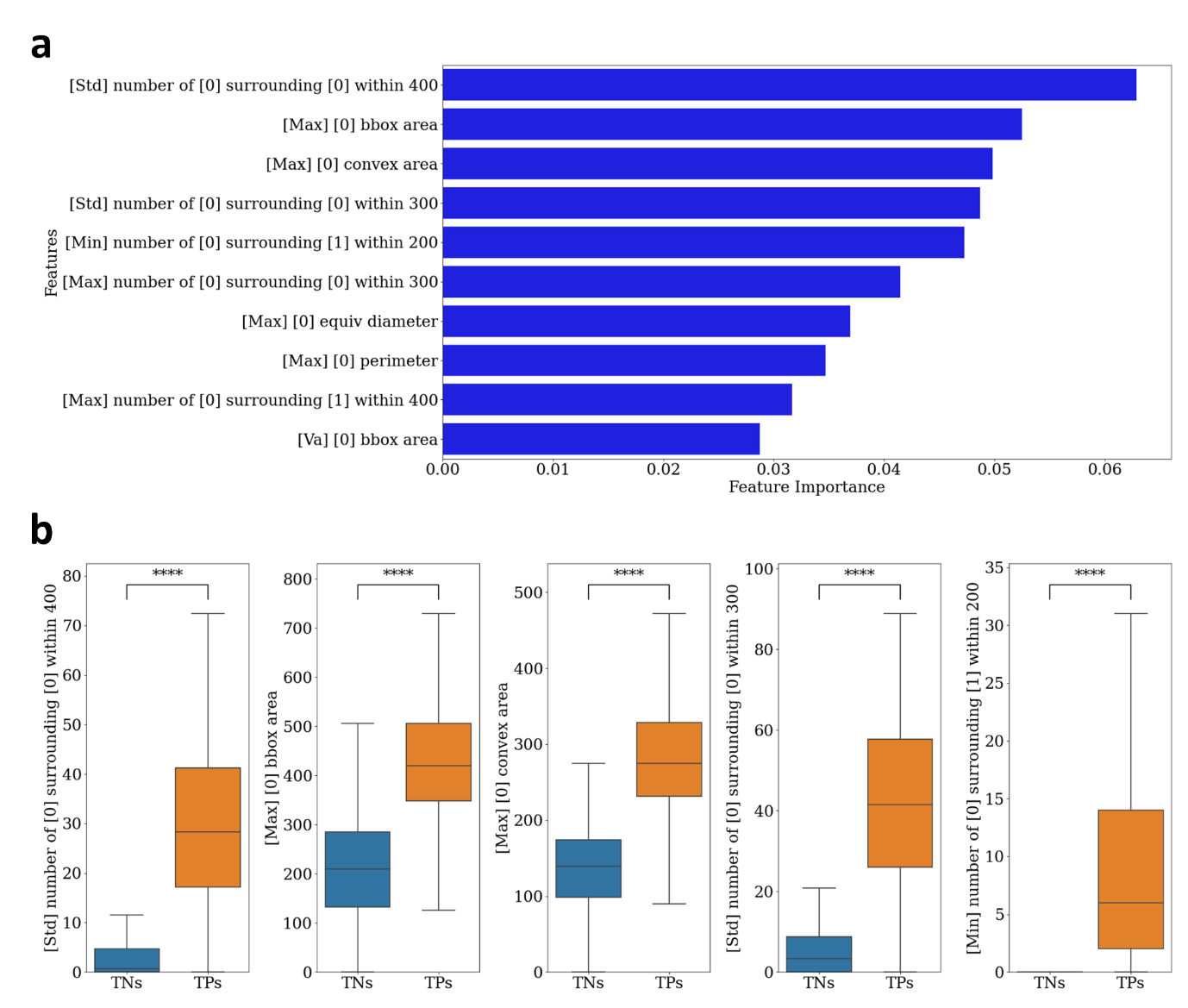


Supplementary Figure 7 a The top ten most important morphological/spatial features found via a Random Forest, and b boxplots showing the distribution of the top five of these features, on external validation. [0] are “other” nuclei, whilst [1] are epithelial nuclei. Distance is measure in microns.


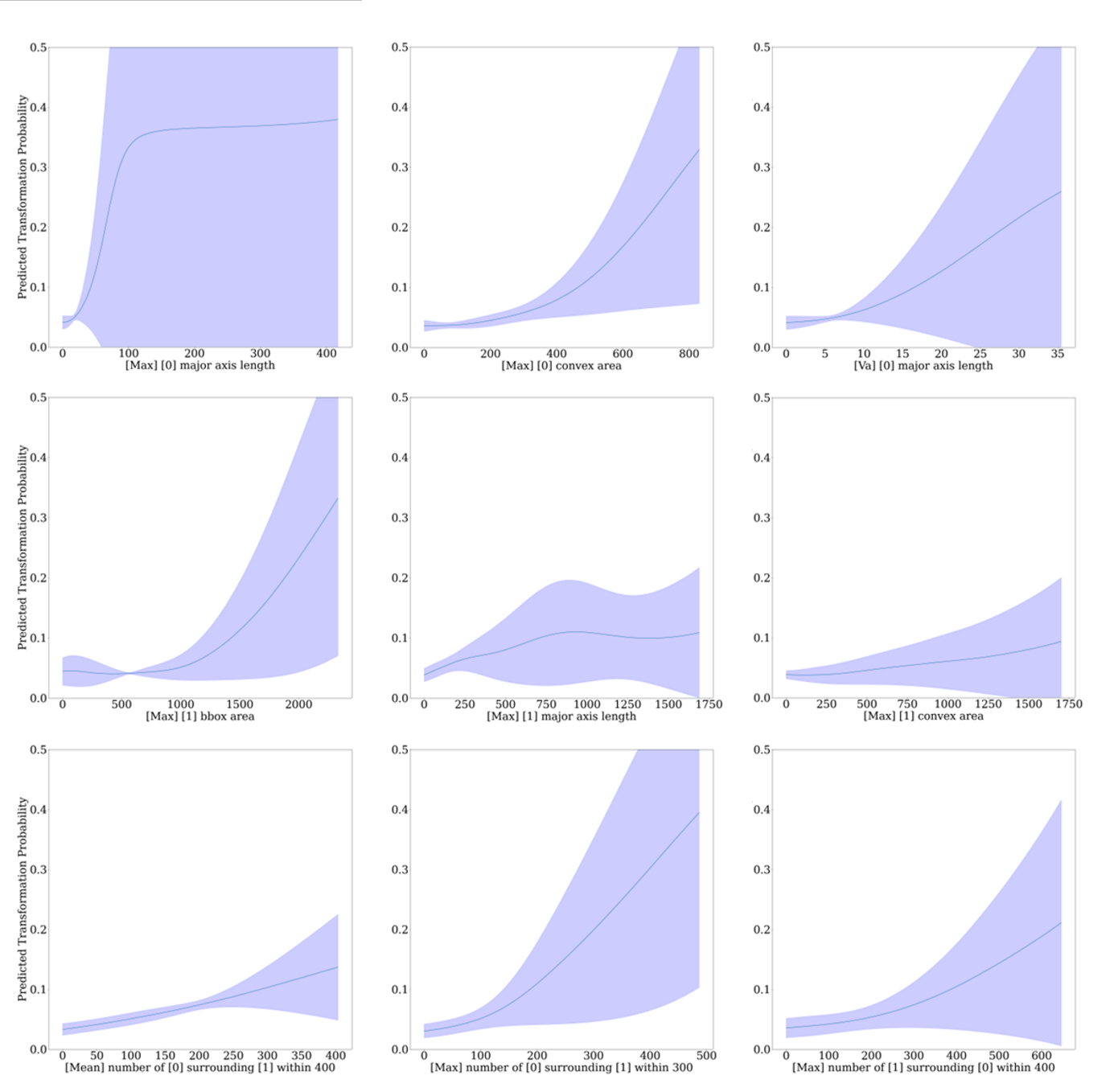


Supplementary Figure 8 Partial Dependency Plots for the *OMTscore* on internal validation. Partial Dependency Probability Plots are given for the *OMTscore* based on the internal validation data. Here, “epithelial” nuclei are labelled as [1] and “other” nuclei are labelled as [0]. Distances are given in microns. The confidence intervals are based on the standard deviation across the three repeats of internal cross-validation experiments.


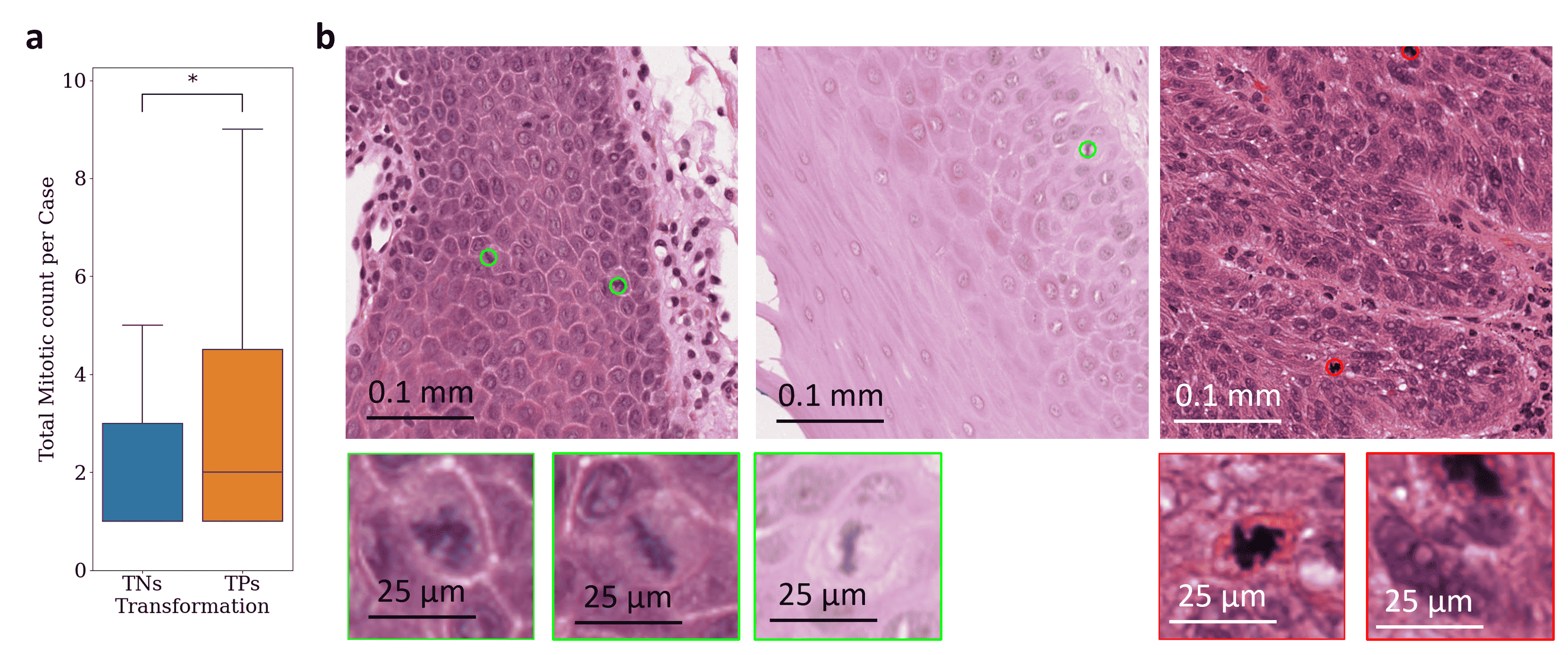


Supplementary Figure 9 Mitotic figure counting. a Mitotic figure counts (MIDOG 2022 winning algorithm), in the top five predicted patches from true positive cases against the top five predicted patches from true negative cases. b Detections with algorithm correctly detected mitoses (green) and incorrectly missed mitoses (red).

Supplementary Figure 10 Kaplan-Meier transformation-free survival curves for the Sheffield and Birmingham-Belfast datasets.


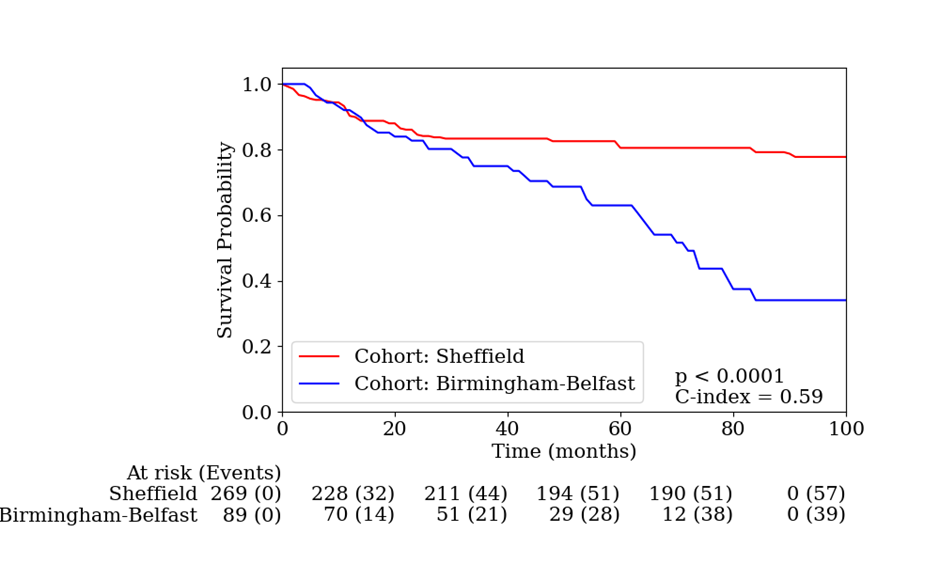

Supplement: Supplementary file 2 — Supplementary Material [file 41698_2024_624_MOESM2_ESM.docx]
